# Associations of plasma level of soluble LDL receptor with cardiovascular events and mortality in a large prospective cohort study

**DOI:** 10.1186/s12944-026-02920-7

**Published:** 2026-04-10

**Authors:** Shieon Kim, Hee Ju Jun, Garam Jo, Dahyun Park, Soyoung Kwak, Min-Jeong Shin, Ronald M. Krauss

**Affiliations:** 1 Interdisciplinary Program in Precision Public Health, Graduate School of Korea University, Seoul, South Korea; 2 Institute for Bio Materials, Korea University, Seoul, South Korea; 3 Department of Food and Nutrition, College of Life Science and Nano Technology, Hannam University, Yuseong-gu, Daejeon, South Korea; 4 Department of Population Health, NYU Grossman School of Medicine, New York, NY USA; 5 NYU Laura and Isaac Perlmutter Cancer Center, New York, NY USA; 6 Department of Microbiology and Immunology, School of Dentistry, Kyungpook National University, Daegu, South Korea; 7 School of Biosystems and Biomedical Sciences, College of Health Science, Korea University, Seoul, South Korea; 8 Departments of Pediatrics and Medicine, University of California, San Francisco, CA USA

**Keywords:** Soluble LDL Receptor (sLDLR), Myocardial Infarction (MI), Heart Failure (HF), Mortality, UK Biobank

## Abstract

**Background:**

Soluble low-density lipoprotein receptor (sLDLR) is proteolytically cleaved from cell membranes and released into the circulation. Prior studies have linked plasma sLDLR to atherogenic lipids, but prospective evidence for its relation to cardiovascular outcomes is limited. This study aimed to evaluate associations between circulating sLDLR and risks of myocardial infarction (MI), heart failure (HF), and cardiovascular (CVD) mortality, as well as all-cause mortality.

**Methods:**

We analyzed data from 47,518 participants in the UK Biobank who were free of CVD at baseline, with a median follow-up of 12.8 years. Nested Cox proportional hazards models were used to estimate associations between Olink-based sLDLR levels and outcomes.

**Results:**

Participants in the highest tertile of sLDLR were older, had higher BMI, and displayed more adverse lipid and inflammatory profiles. In models that included demographic and clinical data, each 1-SD increase of sLDLR was linked to higher MI risk but to lower risk of CVD mortality. With further adjustment for BMI, the association of sLDLR with MI remained significant (HR 1.32, [95% CI 1.17,1.50]), the inverse association with CVD mortality was further strengthened (HR 0.76 [0.64–0.90]), and significant inverse associations with HF and all-cause mortality also became apparent. Notably, HRs for all endpoints except CVD mortality were reduced to insignificance by adjustment for LDL-cholesterol (LDL-C). Time-dependent ROC analyses indicated consistent predictive performance across outcomes for up to 10 years of follow-up.

**Conclusions:**

sLDLR was associated with LDL-C-dependent MI risk but with protection from CVD mortality, and with LDL-C- and BMI-dependent inverse associations with HF and all-cause mortality. These findings point to sLDLR as a potential outcome-specific biomarker and indicate that LDL-C level and adiposity influence the observed associations.

**Supplementary Information:**

The online version contains supplementary material available at 10.1186/s12944-026-02920-7.

## Background

Soluble low-density lipoprotein receptor (sLDLR) consists of the carboxyterminal ligand-binding ectodomain of the low-density lipoprotein receptor (LDLR) that is generated by proteolytic cleavage of LDLR at the cell membrane and released into the circulation [1, 2]. Proteases that have been reported to be responsible for sLDLR production include matrix metalloproteinase-14 (MMP-14, aka MT1-MMP) [2], bone morphogenetic protein-1 (BMP-1) [3], and a disintegrin and metalloprotease 17 (ADAM17) [4].

A relationship of sLDLR to risk of ischemic heart disease (IHD) has been suggested by its correlation with circulating levels of triglycerides (TG) and low-density lipoprotein cholesterol (LDL-C) [5–8], as well as the observation that diet-induced changes in sLDLR concentration were significantly associated with changes in TG, apolipoprotein B (ApoB), and small LDL particles, features of the atherogenic dyslipidemia that characterizes the metabolic syndrome [9]. Moreover, in a recent case-control study, increased plasma sLDLR concentration was associated with incident myocardial infarction (MI) [10], though this was based on a relatively small number of MI cases (*n* = 292), with limited follow-up (≤ 7 years), and without a time-to-event analysis. Beyond IHD, observational data in patients with heart failure (HF) have indicated that higher levels of circulating LDLR were associated with improved survival and lower mortality risk [11]. These findings suggest that sLDLR may play a dual and outcome-specific role in cardiovascular disease (CVD), with pro-atherogenic associations in the context of MI but potentially protective associations for HF and mortality. However, these studies were based on relatively small cohorts with limited follow-up, and thus require confirmation in larger, prospective studies.

In this study, data from the UK Biobank (UKB), a large-scale, long-term prospective cohort, were used to investigate the prospective associations of plasma sLDLR with major CVD outcomes, including incident MI, incident HF, and CVD mortality, as well as all-cause mortality, within a single cohort using a shared analytical approach. This allows for comparison of effect sizes and directions for these outcomes, and extends previous research by assessing mortality outcomes.

## Methods

### UKB Study population

UKB is a prospective cohort built by recruiting nearly half a million adults aged 40 to 69 years across the United Kingdom between 2006 and 2010, with study design and data collection procedures as detailed elsewhere [12]. sLDLR data were generated through the UKB Pharma Proteomics Project (UKB-PPP) in a subsample of 51,644 of UKB participants. Of these, we excluded participants with a history of IHD, HF, stroke, or major neurodegenerative disease (Alzheimer’s disease, Parkinson’s disease, vascular dementia), leaving 47,518 participants for the final analysis (Fig S1) with a mean follow-up of 12.8 years. During the follow-up period, incident events included 1,275 cases of MI, 1,638 cases of HF, 761 CVD deaths, and 4,355 deaths from all causes. Registry data provided clinical information regarding demographics (age, sex, ethnicity, income, education), traditional cardiovascular risk factors (body mass index [BMI], smoking history, alcohol consumption, metabolic equivalent of task [MET] score, diabetes, hypertension) and CVD medication use (statins, angiotensin-converting enzyme inhibitors, angiotensin II receptor blockers, aspirin) [13].

### Measurement of biomarker levels in UKB

Participants included in the UKB-PPP had venous blood drawn at the baseline visit, and the blood was stored at -80 °C until analysis. Plasma levels of sLDLR were measured by proteomics-based quantitative analysis utilizing the Olink platform’s Proximity Extension Assay (PEA) technology [14]. The results were expressed as Normalized Protein Expression (NPX) units provided by Olink, and log-transformed NPX values were used for statistical analysis as described previously [15]. TG, LDL-C, high-density lipoprotein cholesterol (HDL-C), apolipoprotein (Apo) B and A1, C-reactive protein (CRP), and lipoprotein(a) [Lp(a)] were measured using routine blood chemistry assays in UKB [16]. Plasma tumor necrosis factor-alpha (TNF-α) and apolipoprotein E (ApoE) levels were assessed using the Olink proteomics platform. Although levels of other cytokines such as Interleukin-6 were available, only CRP and TNF-α showed significant associations with sLDLR after multiple testing correction and were therefore included as covariates.

### Follow-up and outcomes in UKB

Participant data from the UKB was linked to primary care records and Hospital Episode Statistics from 1997 (England), 1998 (Wales), and 1981 (Scotland) through September 2023, providing a complete history of hospital use over the entire period. Disease events were identified according to International Classification of Diseases, Tenth Revision (ICD-10) codes, based on hospital discharge records and national death registry data, with MI defined as I21 and HF as I50 [17]. The follow-up period was from baseline to the date of death or October 30, 2022, whichever was earlier. The end date of follow-up was based on the latest death registration information from the UKB available at the time of data extraction, with death information obtained from the UK National Death Register. The UK Death Registry was used to ascertain outcomes of CVD mortality and all-cause mortality, as well as to determine the censoring date for participants who died. CVD mortality was defined based on death registry records as deaths with an underlying ICD-10 code starting with “I” (I00-I99). Incident MI and HF events were not ascertained using the death registry, but were identified exclusively from hospital admission records through ICD-10 codes.

### Statistical analysis

Baseline characteristics of participants were summarized using descriptive statistics, with categorical variables presented as counts and percentages and continuous variables as means and standard deviations. Comparisons between groups were made using Pearson chi-square tests for categorical variables and one-way analysis of variance (ANOVA) for continuous variables. The risk of MI events according to sLDLR levels (1 standard deviation increase) was analyzed using nested Cox proportional hazards regression models. sLDLR was standardized as a continuous variable, and hazard ratios (HRs) with 95% confidence intervals (CIs) were estimated using four models with stepwise covariate adjustment. Directed acyclic graph (DAG) analysis was used to further assess the associations of sLDLR, lipids, BMI, and with clinical outcomes and to aid in covariate selection (Fig S2). Due to the potential for BMI to be either in the causal pathway and/or a collider variable, we also utilized models adjusting for BMI and interpreted these as assessing conditional associations. In secondary analyses we further adjusted for lipids, apolipoproteins, and inflammatory markers. The number of samples and events differed between models due to missing covariate information as shown in Table S1. We also performed a sensitivity analysis addressing the possible effects of missingness-related sample loss between Model 2 versus Models 3 and 4 by repeating Models 3 and 4 without income and MET score which were the main contributors to missingness (Table S2).

Subgroup analysis was performed by dividing participants into tertiles according to baseline BMI with an equal number of individuals in each group. Within each tertile, Cox proportional hazards models were applied to examine the associations of serum sLDLR with MI, HF, CVD mortality, and all-cause mortality. We modeled sLDLR using restricted cubic splines (RCS; 4 knots) in Cox models, tested for non-linearity with a Wald test, and plotted adjusted spline-based HR curves (95% CIs) using a pre-specified reference. We also performed a positive predictive value (PPV)-based bias analysis for MI and HF using PPVs from UK Biobank validation studies (MI ≈ 0.80; HF ≈ 0.70) [18, 19] with 1,000 simulations.

To evaluate the discriminatory power of the models, time-dependent receiver operating characteristic curves (ROC curves) at 5 and 10 years were generated from Model 4 for each outcome. The total data were randomly split into training and test sets in a 70:30 ratio [20], and the area under the curve (AUC) was calculated for the test set. Reclassification analyses were prioritized for MI as the endpoint most closely aligned with atherosclerotic CVD risk [21]. We computed category-free NRI and IDI for 10-year MI risk comparing the base clinical model versus the base model plus sLDLR, excluding prevalent MI at baseline, with 95% CIs obtained by bootstrap (500 replications). All analyses were performed using Stata version 17.0 (StataCorp, College Station, TX, USA) and Python 3.10 executed in the Google Collaboratory environment.

## Results

### Baseline characteristics and lipid correlates of sLDLR in the UKB cohort

Participants were divided into tertiles of sLDLR levels, and demographic and clinical baseline characteristics were compared between groups **(**Table 1**)**. Overall, individuals in higher sLDLR tertiles were older (*p* < 0.001), and sex distribution differed significantly, with a higher proportion of men in Tertile 2 and women in Tertile 1 (*p* < 0.001). The proportion of Asian participants was slightly higher across tertiles, whereas the proportion of Black participants was lower (*p* < 0.001). Higher sLDLR tertiles were associated with greater smoking prevalence and slightly less alcohol consumption (both *p* < 0.001). Additionally diabetes and hypertension were more prevalent in the highest tertile (*p* = 0.048 and *p* < 0.001, respectively) and BMI, TG, and LDL-C all increased progressively from Tertile 1 to Tertile 3 (*p* < 0.001 for each). In contrast, HDL-C and ApoA1 levels decreased across tertiles (both *p* < 0.001), whereas ApoB and ApoE levels showed a significant increasing trend. Lp(a), TNF-α, and CRP also progressively increased with higher sLDLR tertiles (*p* < 0.001 for all).

**Table 1:** Baseline Characteristics of Participants According to sLDLR Tertile

Characteristic Mean (SD) or *N* (%)	Tertile 1	Tertile 2	Tertile 3	*p*-value
	*N* = 15,840	*N* = 15,840	*N* = 15,838	
LDLR range	-2.35 to -0.26	-0.26 to 0.26	0.26 to 2.78	
Demographic factors				
Age	54.91 (8.59)	56.93 (8.03)	57.26 (7.81)	**< 0.001**
Sex				**< 0.001**
Male	6,677 (42.2%)	7,430 (46.9%)	6,983 (44.1%)	
Female	9,163 (57.8%)	8,410 (53.1%)	8,855 (55.9%)	
Ethnicity group				**< 0.001**
Asian	264 (1.7%)	329 (2.1%)	412 (2.6%)	
White	14,740 (93.5%)	14,793 (93.9%)	14,742 (93.5%)	
Black	449 (2.8%)	348 (2.2%)	312 (2.0%)	
Others	310 (2.0%)	290 (1.8%)	293 (1.9%)	
Smoking history				**< 0.001**
No	9,228 (58.5%)	8,699 (55.2%)	8,363 (53.1%)	
Yes	6,550 (41.5%)	7,071 (44.8%)	7,385 (46.9%)	
Alcohol drinking history				**< 0.001**
No	613 (3.9%)	727 (4.6%)	843 (5.3%)	
Yes	15,186 (96.1%)	15,079 (95.4%)	14,945 (94.7%)	
Diabetes mellitus	4,006 (25.3%)	4,041 (25.5%)	4,186 (26.4%)	**0.048**
Hypertension	9,390 (63.1%)	9,880 (66.5%)	10,117 (68.3%)	**< 0.001**
Clinical variables				
BMI, Kg/m2	25.45 (4.16)	27.44 (4.54)	29.08 (4.77)	**< 0.001**
Triglycerides, mmol/L	1.091 (0.454)	1.609 (0.670)	2.493 (1.194)	**< 0.001**
LDL-C, mmol/L	3.191 (0.692)	3.616 (0.771)	3.957 (0.925)	**< 0.001**
HDL-C, mmol/L	1.598 (0.404)	1.446 (0.364)	1.333 (0.326)	**< 0.001**
ApoB, g/L	0.920 (0.186)	1.043 (0.205)	1.152 (0.259)	**< 0.001**
ApoA1, g/L	1.597 (0.279)	1.539 (0.267)	1.494 (0.253)	**< 0.001**
ApoE	− 0.030 (0.792)	0.010 (0.720)	0.205 (0.700)	**< 0.001**
Lp(a), nmol/L	43.471 (48.430)	45.540 (49.253)	45.825 (49.702)	**< 0.001**
TNF-α	-0.070 (0.436)	0.023 (0.408)	0.110 (0.420)	**< 0.001**
CRP, mg/L	2.13 (4.63)	2.54 (4.17)	3.10 (4.11)	**< 0.001**

Values are presented as mean (SD) for continuous variables and N (%) for categorical variables. p-values were calculated using ANOVA for continuous variables and Chi-square tests for categorical variables to compare differences across LDLR tertiles. Bolded p-values indicate statistically significant differences (*p* < 0.05)

*Abbreviations: sLDLR *soluble low-density lipoprotein receptor, *BMI *body mass index, *LDL-C* low-density lipoprotein cholesterol, *HDL-C* high-density lipoprotein cholesterol, *ApoB *apolipoprotein B, *ApoA1 *apolipoprotein A1, *ApoE *apolipoprotein E, *Lp(a)* lipoprotein(a), *CRP *C-reactive protein, *TNF*-*α *tumor necrosis factor-alpha

### Associations of circulating sLDLR levels with lipids and inflammatory markers

Circulating sLDLR levels were positively correlated with several lipid and inflammatory markers (Fig S3). Specifically, sLDLR levels were strongly correlated with plasma TG levels (*r* = 0.69, *p* < 0.001) and moderately correlated with LDL-C (*r* = 0.38, *p* < 0.001). In addition, sLDLR was significantly positively correlated with the inflammatory biomarkers CRP (*r* = 0.26, *p* < 0.001) and TNF-α (*r* = 0.19, *p* < 0.001). These results suggest that high sLDLR levels are associated with an atherogenic lipid profile and systemic inflammation.

### Longitudinal association between sLDLR levels and clinical outcomes

**Fig. 1:**
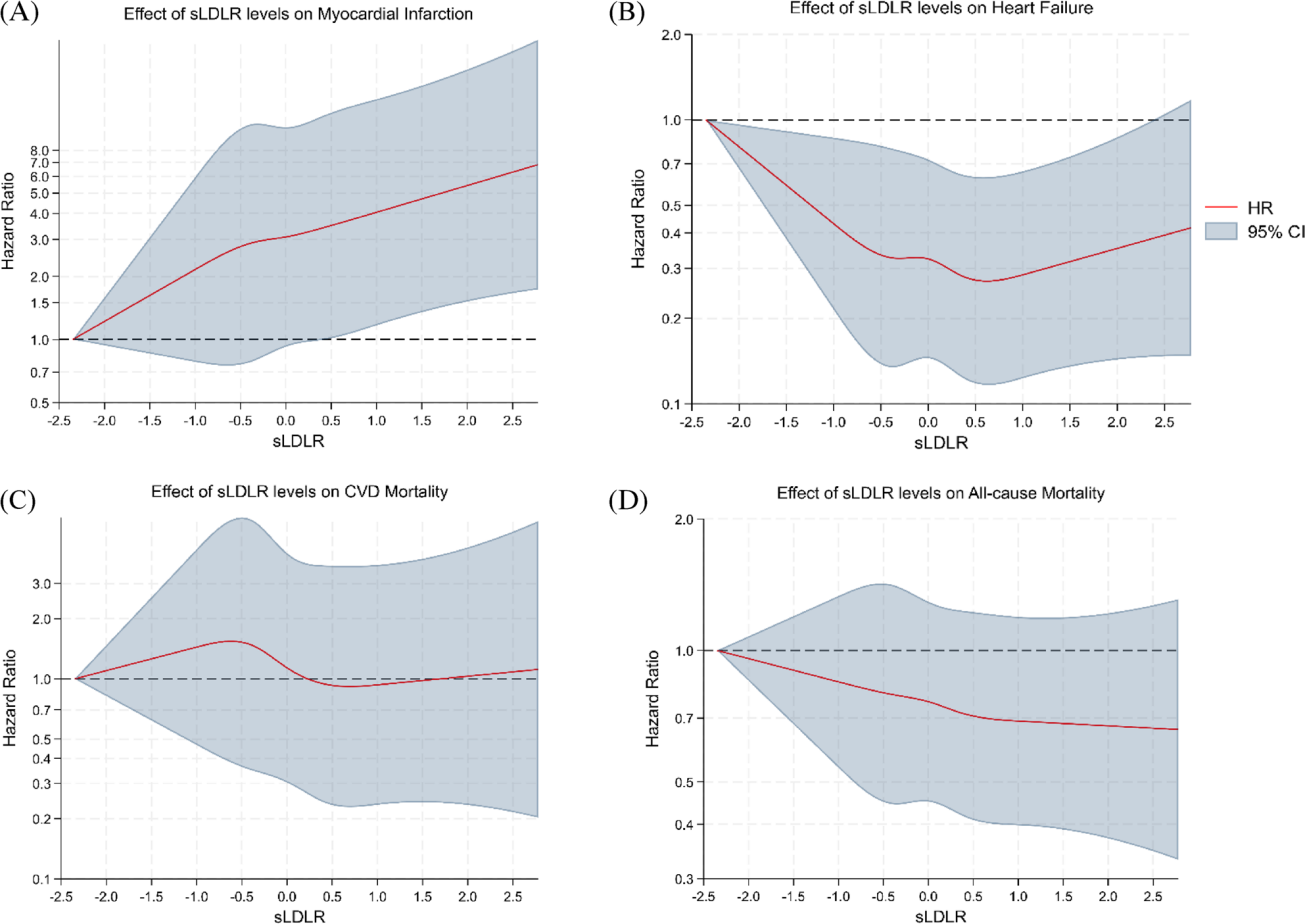
Restricted cubic spline analyses of sLDLR and clinical outcomes. Abbreviations: sLDLR, soluble low-density lipoprotein receptor Restricted cubic splines (4 knots) from multivariable Cox models (Model 4) are shown. The x-axis represents sLDLR levels on the NPX scale. The red line indicates the estimated hazard ratio (HR) across the range of sLDLR, and the shaded area denotes the 95% confidence interval (CI). The horizontal dashed line marks HR = 1.0 (reference). Estimates are presented as BMI-adjusted conditional associations

Quantitation in multivariable Cox proportional hazards models revealed that each 1-SD increase in sLDLR was significantly associated with an increased risk of MI, but with reduced incidence of CVD mortality (Table 2). In models with demographic and clinical variables but without BMI, HRs were close to null for HF and all-cause mortality, but inverse associations with these endpoints were observed after further adjusting for BMI (HR 0.84 [0.75–0.94], *p* = 0.003) and 0.90 [0.84–0.97], *p* = 0.004), respectively. The association of sLDLR with MI remained strong after BMI adjustment (HR 1.32, [95% CI 1.17–1.50], *p* < 0.001), while the inverse association with CVD mortality was strengthened (HR 0.76 [0.64–0.90], *p* = 0.001). BMI-stratified analyses showed that the MI association was most evident at lower levels of BMI and that there were increasingly inverse point estimates for mortality outcomes at higher BMI levels (Fig S4), although tests for interaction were not statistically significant. We further assessed potential non-linearity using RCS in the BMI-adjusted model (Model 4). The spline curves suggested an approximately monotonic increase in MI risk with higher sLDLR, whereas HF and mortality outcomes showed predominantly inverse patterns with wider uncertainty at the extremes (Fig. 1). As a sensitivity analysis addressing outcome misclassification, PPV-based bias analyses for MI and HF yielded virtually unchanged estimates (Table S3).

**Table 2:** Cox proportional hazards model of sLDLR and incident disease risk

	per 1-SD							
	Myocardial Infarction		Heart Failure		CVD mortality		All-cause mortality	
	HR (95% CI)	p	HR (95% CI)	p	HR (95% CI)	p	HR (95% CI)	p
Model 1	1.43 (1.31, 1.56)	**< 0.001**	1.16 (1.07, 1.25)	**< 0.001**	0.98 (0.87, 1.09)	0.680	1.06 (1.01, 1.11)	**0.018**
Model 2	1.39 (1.27, 1.52)	**< 0.001**	1.07 (0.99, 1.16)	0.103	0.90 (0.80, 1.02)	0.090	0.96 (0.91, 1.01)	0.110
Model 3	1.36 (1.21, 1.53)	**< 0.001**	0.98 (0.88, 1.09)	0.714	0.82 (0.70, 0.96)	**0.016**	0.95 (0.89, 1.01)	0.102
Model 4: Model 3 + BMI	1.32 (1.17, 1.50)	**< 0.001**	0.84 (0.75, 0.94)	**0.003**	0.76 (0.64, 0.90)	**0.001**	0.90 (0.84, 0.97)	**0.004**

Model 1: unadjusted; Model 2: adjusted for age, sex, and ethnicity; Model 3: additionally adjusted for education, smoking, drinking, physical activity (MET), diabetes, hypertension, and CVD medication use; the final row shows further adjustment for BMI. Sample sizes reflect complete-case analysis for each model. Bolded p-values indicate statistically significant differences (*p* < 0.05)

*Abbreviations:sLDLR *soluble low-density lipoprotein receptor, *CVD *cardiovascular disease, *BMI *body mass index

As shown in Table 3, after additional adjustment for TG, HDL-C, or ApoA1, the overall directions and significance of associations with sLDLR were largely preserved. By contrast, adjustment for LDL-C reduced the associations with MI, HF, and all-cause mortality to non-significance, while the protective association with CVD mortality remained robustly inverse (HR 0.68, 95% CI 0.56–0.83, *p* < 0.001). Similar trends were observed with adjustment for ApoB (Table 3). Adjustments for inflammatory markers, ApoA1, ApoE, and Lp(a) did not materially alter the results. After excluding events occurring within the first 3 years, effect directions were broadly consistent across models, with some attenuation or shifts in statistical significance in biomarker-adjusted models, likely reflecting reduced events and precision (Table S4). Analyses using sLDLR tertiles yielded consistent patterns, showing higher MI risk but lower risks of HF, CVD mortality, and all-cause mortality across increasing tertiles (Table S5). 

**Table 3:** Extended models with additional adjustment for lipid and inflammatory markers

	per 1-SD							
	*n* = 47,518							
	Myocardial Infarction		Heart Failure		CVD mortality		All-cause mortality	
	HR (95% CI)	*p*	HR (95% CI)	*p*	HR (95% CI)	*p*	HR (95% CI)	*p*
Model 4 + TG	1.28 (1.10, 1.50)	**0.002**	0.81 (0.70, 0.93)	**0.004**	0.67 (0.54, 0.83)	**< 0.001**	0.89 (0.81, 0.97)	**0.010**
Model 4 + LDL-C	1.08 (0.94, 1.25)	0.265	0.88 (0.78, 1.00)	0.050	0.68 (0.56, 0.83)	**< 0.001**	0.94 (0.87, 1.01)	0.089
Model 4 + HDL-C	1.21 (1.06, 1.39)	**0.005**	0.85 (0.75, 0.96)	**0.012**	0.77 (0.64, 0.93)	**0.007**	0.90 (0.83, 0.97)	**0.006**
Model 4 + TG, LDL-C	1.06 (0.89, 1.26)	0.493	0.83 (0.71, 0.97)	**0.019**	0.61 (0.48, 0.77)	**< 0.001**	0.91 (0.83, 1.00)	0.054
Model 4 + TG, LDL-C, HDL-C	0.99 (0.82, 1.20)	0.949	0.86 (0.73, 1.01)	0.062	0.61 (0.48, 0.78)	**< 0.001**	0.91 (0.83, 1.00)	0.062
Model 4 + ApoB	1.06 (0.91, 1.22)	0.448	0.86 (0.76, 0.98)	**0.019**	0.67 (0.55, 0.81)	**< 0.001**	0.91 (0.85, 0.99)	**0.025**
Model 4 + ApoA1	1.23 (1.08, 1.40)	**0.002**	0.85 (0.76, 0.97)	**0.013**	0.76 (0.63, 0.92)	**0.004**	0.90 (0.84, 0.97)	**0.008**
Model 4 + ApoE	1.33 (1.16, 1.53)	**< 0.001**	0.82 (0.73, 0.93)	**0.003**	0.73 (0.61, 0.89)	**0.002**	0.91 (0.84, 0.99)	**0.019**
Model 4 + Lp(a)	1.38 (1.21, 1.59)	**< 0.001**	0.84 (0.74, 0.96)	**0.012**	0.72 (0.59, 0.88)	**0.001**	0.90 (0.83, 0.98)	**0.014**
Model 4 + TNFa	1.30 (1.15, 1.47)	**< 0.001**	0.83 (0.74, 0.93)	**0.002**	0.73 (0.62, 0.87)	**< 0.001**	0.87 (0.81, 0.94)	**< 0.001**
Model 4 + CRP	1.28 (1.13, 1.45)	**< 0.001**	0.83 (0.74, 0.94)	**0.002**	0.73 (0.61, 0.87)	**< 0.001**	0.89 (0.83, 0.95)	**0.001**

Analyses were based on Model 4 (adjusted for age, sex, ethnicity, education, smoking status, alcohol consumption, physical activity [MET], diabetes mellitus, hypertension, cardiovascular medication use, and BMI), with additional adjustments for the variables indicated. Sample sizes reflect complete-case analysis, and CRP levels were log-transformed. Bolded p-values indicate statistically significant differences (*p* < 0.05)

*Abbreviations*: *TG *triglycerides, *LDL-C* low-density lipoprotein cholesterol, *HDL-C* high-density lipoprotein cholesterol, *ApoB *apolipoprotein B, *ApoA1 *apolipoprotein A1, *ApoE *apolipoprotein E, *Lp*(*a*) lipoprotein(a), *CRP *C-reactive protein, *TNF*-*α* tumor necrosis factor-alpha, *BMI *body mass index

### Time-dependent predictive accuracy of sLDLR

**Fig. 2:**
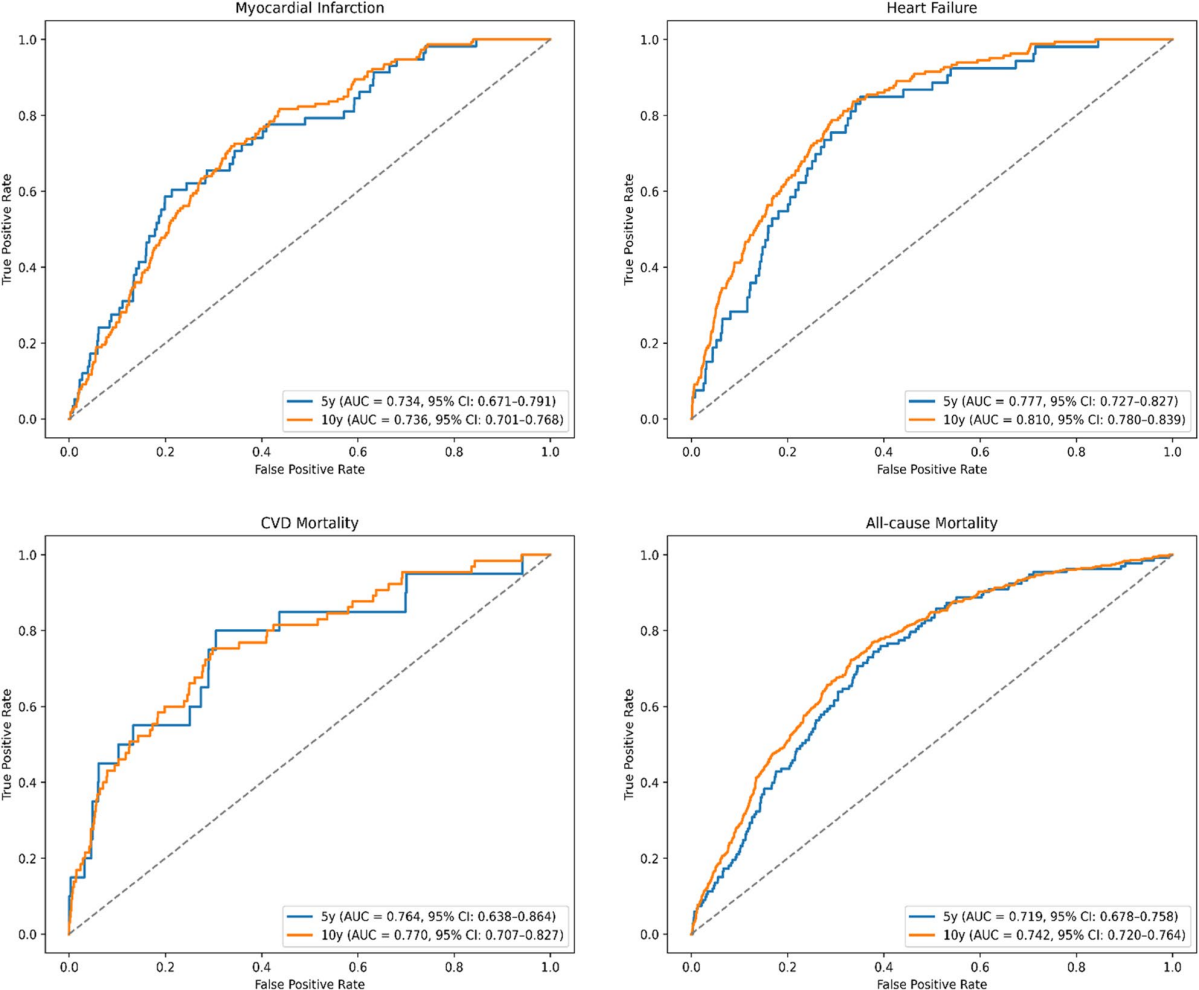
Time-dependent ROC curves for predicting disease outcomes based on circulating sLDLR levels

To further assess the predictive ability of sLDLR for different outcomes, time-dependent ROC analyses were carried out using Cox proportional hazards based on model 4, which included adjustment for BMI (Fig. 2). For incident MI, the AUC was 0.734 at 5 years (95% CI: 0.671–0.791) and 0.736 at 10 years (95% CI: 0.701–0.768). For incident HF, the corresponding values were 0.777 at 5 years (95% CI: 0.727–0.827) and 0.810 at 10 years (95% CI: 0.780–0.839). For CVD mortality, the AUC reached 0.764 at 5 years (95% CI: 0.638–0.864) and 0.770 at 10 years (95% CI: 0.707–0.827). Finally, for all-cause mortality, the AUC was 0.719 at 5 years (95% CI: 0.678–0.758) and 0.740 at 10 years (95% CI: 0.720–0.764). The number of events and censored observations at each time point are presented in the Table S6, indicating increasing event rates and decreasing censoring over time.

For incident MI, adding sLDLR improved category-free NRI in both Model 3 (0.113, 95% CI 0.021–0.205; *p* = 0.017) and Model 4 (0.094, 0.007–0.182; *p* = 0.035), whereas IDI was not significant (Model 3: 0.00095, *p* = 0.101; Model 4: 0.00085, *p* = 0.112). These results indicate that discrimination was stable across outcomes, and that MI risk reclassification improved with addition of sLDLR.

## Discussion

In this large prospective analysis in the UKB with a median follow-up of 12.8 years, we found that circulating sLDLR levels showed differential associations with cardiovascular outcomes and CVD and all-cause mortality. Quantitative Cox modeling revealed that each 1-SD increase in sLDLR was significantly associated with a higher risk of MI, while at the same time predicting lower risks of CVD mortality, and inverse associations of sLDLR with HF and all-cause mortality became evident only after BMI adjustment.

We observed that elevated sLDLR levels were correlated with increased TG and LDL-C, as well as the inflammatory factors CRP and TNF-α, consistent with previous findings [5–9]. For MI, the adverse association with higher sLDLR was reduced to insignificance after adjusting for LDL-C or ApoB, but was not substantially affected by adjustment for the inflammatory markers. This supports the notion that the observed MI risk is primarily mediated by correlations with atherogenic lipoproteins. Mechanistic evidence supports this interpretation. sLDLR can bind LDL, thereby inhibiting its hepatic uptake and leading to greater arterial exposure [10]. Similarly, binding of sLDLR to triglyceride-rich VLDL particles may delay their clearance [2], linking sLDLR with both elevated plasma TG and increased atherogenic risk. Both hepatic MT1-MMP overexpression [2] and exposure to inflammatory cytokines [10] have been shown to promote sLDLR shedding, leading to reduced cellular LDLR and higher plasma LDL-C and non-HDL-C levels [2]. Conversely, inhibition of MT1-MMP-mediated LDLR proteolysis in murine models reduces plasma cholesterol and attenuates atherosclerotic lesion progression [2]. These findings suggest that sLDLR contributes to MI risk via effects on atherogenic lipoprotein levels.

Because adiposity is tightly linked to both lipid metabolism and clinical prognosis, the role of BMI in the sLDLR-outcome associations is inherently complex. In our data, inverse relationships of with HF and all-cause mortality were seen only after adjusting for BMI, and both became insignificant after further adjustment for LDL-C. While BMI and LDL metabolism could plausibly be considered to be in the causal pathways for the relationship between sLDLR and these clinical outcomes, we consider these relationships to be conditional, rather than indicating a true protective effect of sLDLR. For BMI, this is consistent with previous literature indicating non-linear, often paradoxical, relationships between adiposity and the prognosis of HF, often referred to as the ‘obesity paradox’ [22].

To further explore these BMI-dependent findings, we provide stratified analyses by BMI category and by use of DAG. In stratified analyses, point estimates were consistent with the MI outcome, showing the strongest association with MI at lower BMI levels, while inverse point estimates for mortality were more pronounced at higher BMI levels, although tests for interaction were non-statistically significant. An important point is that the association between sLDLR and incident HF did not vary substantially by BMI category, which may indicate that any effect of BMI is more relevant to survival outcomes rather than to the onset of HF. Future studies with adjudicated HF phenotypes and repeated measures of sLDLR will be necessary to determine whether sLDLR is related to the pathogenesis versus prognosis of HF.

The basis for the observed BMI-conditional patterns for mortality remains uncertain. Beyond biological heterogeneity, differences in underlying health status and subclinical disease, which correlate with both BMI and survival, may contribute to conditioning-related effects in observational cohorts. In addition, misclassification of cause of death could influence associations for non-CVD mortality. Taken together, these considerations underscore the need to interpret the BMI-adjusted estimates cautiously and to validate these findings in independent cohorts with more diverse populations and adjudicated outcomes. Mechanistically, adiposity-related differences in lipid handling and tissue lipotoxic stress have been proposed as potential contributors to clinical outcome heterogeneity [23], but such explanations remain speculative and warrant mechanistic investigation.

Strengths of this study are its use of a large, well-characterized population-based cohort and the long-term evaluation of diverse cardiovascular outcomes. There are however several limitations. The UKB cohort consists predominantly of white, middle-aged individuals, which may limit the generalizability of our findings. and sLDLR levels were measured at a single time point, precluding assessment of temporal variability [24]. Moreover, as with any observational study, causal relationships cannot be definitively established [25]. It is possible for example that some or all of the observed associations are not due to sLDLR itself, but to other factors with which sLDLR may be correlated. In addition, Olink PEA measures an LDLR-derived circulating fragment, but without epitope mapping or orthogonal validation (e.g., western blot/mass spectrometry), and therefore we cannot confirm that this measurement specifically or preferentially reflects the functional ligand-binding ectodomain, thus further limiting mechanistic interpretation. Furthermore, within-person and pre-analytical variation, as well as long-term analyte stability in UKB-PPP samples stored for ≥ 10 years, were not characterized [26]. Blinded duplicate participant samples were also not available in our analytic dataset to estimate measurement reliability (e.g., ICC), and any non-differential measurement error could attenuate effect estimates and weaken interaction tests. Additionally, baseline subclinical cardiovascular disease (e.g., coronary calcification or impaired LV function) could increase sLDLR shedding via underlying endothelial inflammation, raising the possibility of reverse causation and further limiting causal interpretation. Finally, since the data for the present study were derived from the UKB-PPP analytic sample, confirmation of the results in broader and more diverse populations will be needed to determine their generalizability.

## Conclusions

This large prospective study demonstrates that circulating sLDLR levels are associated with an LDL-dependent increase in risk of MI but a reduced risk of CVD mortality, as well as BMI-related protection from HF and total mortality. Together, the findings position sLDLR as a potential biomarker for diverse pathways that differentially impact risk of MI and related disease outcomes, warranting further mechanistic and clinical investigation.

## Supplementary Information

Supplementary Material 1.

Supplementary Material 2.

## Data Availability

The data that support the findings of this study were obtained from the UK Biobank under an approved application and are not publicly available due to access restrictions. Data may be available from the authors upon reasonable request and with permission from the UK Biobank.

## Abbreviations

ADAM17: A Disintegrin and Metalloprotease 17
ANOVA: One-way analysis of variance
ApoA1: Apolipoprotein A1
ApoB: Apolipoprotein B
ApoE: Apolipoprotein E
AUC: Area under the curve
BMI: Body mass index
BMP-1: Bone morphogenetic protein-1
Chr: Chromosome
CI: Confidence interval
CRP: C-reactive protein
CVD: Cardiovascular disease
DAG: Directed acyclic graph
DM: Diabetes mellitus
HDL-C: High-density lipoprotein cholesterol
HF: Heart failure
HR: Hazard ratio
IDI: Integrated discrimination improvement
IHD: Ischemic heart disease
LDL-C: Low-density lipoprotein cholesterol
LDLR: Low-density lipoprotein receptor
Lp(a): Lipoprotein(a)
MET: Metabolic equivalent of task
MI: Myocardial infarction
MMP-14: Matrix metalloproteinase-14 (also known as MT1-MMP)
NPX: Normalized Protein Expression
NRI: Net reclassification improvement
PPV: Positive predictive value
RCS: Restricted cubic spline(s)
ROC: Receiver operating characteristic
SD: Standard deviation
SE: Standard error
sLDLR: Soluble low-density lipoprotein receptor
TG: Triglycerides
TNF-α: Tumor necrosis factor-alpha
UKB: UK Biobank

## References

[1] Fischer DG, Tal N, Novick D, Barak S, Rubinstein M. An Antiviral Soluble Form of the LDL Receptor Induced by Interferon. Science. 1993;262(5131):250–3. 10.1126/science.8211145. 8211145 10.1126/science.8211145

[2] Alabi A, Xia XD, Gu HM, et al. Membrane type 1 matrix metalloproteinase promotes LDL receptor shedding and accelerates the development of atherosclerosis. Nat Commun. 2021;12:1889. 10.1038/s41467-021-22167-3. 33767172 10.1038/s41467-021-22167-3 PMC7994674

[3] Banerjee S, Andrew RJ, Duff CJ, et al. Proteolysis of the low density lipoprotein receptor by bone morphogenetic protein-1 regulates cellular cholesterol uptake. Sci Rep. 2019;9(1):11416. 10.1038/s41598-019-47814-0. 31388055 10.1038/s41598-019-47814-0 PMC6684651

[4] Strøm TB, Tveten K, Laerdahl JK, Leren TP. Mutation G805R in the transmembrane domain of the LDL receptor gene causes familial hypercholesterolemia by inducing ectodomain cleavage of the LDL receptor in the endoplasmic reticulum. FEBS Open Bio. 2014;4:321–7. 10.1016/j.fob.2014.03.007. 24918045 10.1016/j.fob.2014.03.007 PMC4048843

[5] Mbikay M, Mayne J, Chrétien M. The enigma of soluble LDLR: could inflammation be the key? Lipids Health Dis. 2020;19(1):17. 10.1186/s12944-020-1199-9. 32014013 10.1186/s12944-020-1199-9 PMC6998292

[6] Mayne J, Ooi TC, Tepliakova L, et al. Associations Between Soluble LDLR and Lipoproteins in a White Cohort and the Effect of PCSK9 Loss-of-Function. J Clin Endocrinol Metab. 2018;103(9):3486–95. 10.1210/jc.2018-00777. 29982529 10.1210/jc.2018-00777

[7] Shimohiro H, Taniguchi SI, Koda M, Sakai C, Yamada S. Association Between Serum Soluble Low-Density Lipoprotein Receptor Levels and Metabolic Factors in Healthy Japanese Individuals. J Clin Lab Anal. 2015;29(1):52–6. 10.1002/jcla.21727. 24687274 10.1002/jcla.21727 PMC6807053

[8] Girona J, Rodríguez-Borjabad C, Ibarretxe D, et al. Plasma inducible degrader of the LDLR, soluble low-density lipoprotein receptor, and proprotein convertase subtilisin/kexin type 9 levels as potential biomarkers of familial hypercholesterolemia in children. J Clin Lipidol. 2018;12(1):211–8. 10.1016/j.jacl.2017.10.003. 29102496 10.1016/j.jacl.2017.10.003

[9] Krauss RM, Fisher LM, King SA, Gardner CD. Changes in soluble LDL receptor and lipoprotein fractions in response to diet in the DIETFITS weight loss study. J Lipid Res. 2024;65(3):100503. 10.1016/j.jlr.2024.100503. 38246235 10.1016/j.jlr.2024.100503 PMC10882123

[10] Zegeye MM, Nakka SS, Andersson JSO, et al. Soluble LDL-receptor is induced by TNF-α and inhibits hepatocytic clearance of LDL-cholesterol. J Mol Med. 2023;101(12):1615–26. 10.1007/s00109-023-02379-4. 37861809 10.1007/s00109-023-02379-4 PMC10697900

[11] Bayes–Genis A, Núñez J, Zannad F, Ferreira JP, Anker SD, Cleland JG, et al. The PCSK9–LDL receptor axis and outcomes in heart failure: BIOSTAT–CHF subanalysis. J Am Coll Cardiol. 2017;70(17):2128–36. 10.1016/j.jacc.2017.08.057. 29050560 10.1016/j.jacc.2017.08.057

[12] Bycroft C, Freeman C, Petkova D, et al. The UK Biobank resource with deep phenotyping and genomic data. Nature. 2018;562(7726):203–9. 10.1038/s41586-018-0579-z. 30305743 10.1038/s41586-018-0579-z PMC6786975

[13] Tian F, Chen L, Qian Z (Min), editors. Ranking age-specific modifiable risk factors for cardiovascular disease and mortality: evidence from a population-based longitudinal study. *eClinicalMedicine*. 2023;64:102230. 10.1016/j.eclinm.2023.102230 10.1016/j.eclinm.2023.102230 PMC10626167 37936651

[14] Sun BB, Chiou J, Traylor M et al. Genetic regulation of the human plasma proteome in 54,306 UK Biobank participants. Published online June 18, 2022:2022.06.17.496443. 10.1101/2022.06.17.496443

[15] Yadalam AK, Gold ME, Patel KJ et al. Proteomics-Based Soluble Urokinase Plasminogen Activator Receptor Levels Are Associated With Incident Heart Failure Risk. JACC Adv. 2025;4:101442. https://pmc.ncbi.nlm.nih.gov/articles/PMC11683231/. 10.1016/j.jacadv.2024.101442 PMC11683231 39737138

[16] Welsh C, Celis-Morales CA, Brown R, et al. Comparison of Conventional Lipoprotein Tests and Apolipoproteins in the Prediction of Cardiovascular Disease. Circulation. 2019;140(7):542–52. 10.1161/CIRCULATIONAHA.119.041149. 31216866 10.1161/CIRCULATIONAHA.119.041149 PMC6693929

[17] Wolf S, Schievano E, Amidei CB, et al. Mortality trend of ischemic heart disease (2008–2022): A retrospective analysis of epidemiological data. Int J Cardiol. 2024;406:132042. 10.1016/j.ijcard.2024.132042. 38614362 10.1016/j.ijcard.2024.132042

[18] SchnierC, Bush K, Nolan J, Sudlow C, on behalf of the UK Biobank Outcome Adjudication Group. Definitions of acute myocardial infarction and main myocardial infarction pathological types: UK Biobank phase 1 outcomes adjudication [Internet]. August 2017 [cited 2026 Mar 9]. Available from: UK Biobank Showcase. https://biobank.ndph.ox.ac.uk/showcase/showcase/docs/alg_outcome_mi.pdf.

[19] Davidson J, Banerjee A, Muzambi R, Smeeth L, Warren-Gash C. Validity of acute cardiovascular outcome diagnoses recorded in European electronic health records: a systematic review. Clin Epidemiol. 2020;12:1095–111. 33116903 10.2147/CLEP.S265619 PMC7569174

[20] Ramos-Pollán R, Guevara-López MÁ, Oliveira E, Introducing ROC. Curves as Error Measure Functions: A New Approach to Train ANN-Based Biomedical Data Classifiers. In: Bloch I, Cesar RM, editors. Progress in Pattern Recognition, Image Analysis, Computer Vision, and Applications. Springer; 2010. pp. 517–24. 10.1007/978-3-642-16687-7_68.

[21] Goff DC Jr, Lloyd-Jones DM, Bennett G, Coady S, D’Agostino RB, Sr, Gibbons R, et al. 2013 ACC/AHA guideline on the assessment of cardiovascular risk: a report of the American College of Cardiology/American Heart Association Task Force on Practice Guidelines. Circulation. 2014;129(25 Suppl 2):S49–73. 10.1161/01.cir.0000437741.48606.98. 24222018 10.1161/01.cir.0000437741.48606.98

[22] Wang Y, Liu X, Wu B, Tan X, Chen L, Chu H, et al. Mediation effect analysis of lipoprote in levels on BMI and cardiovascular outcomes in patients with heart failure. BMC Cardiovasc Disord. 2024;24(1):553. 10.1186/s12872-024-04155-9. 39395939 10.1186/s12872-024-04155-9 PMC11470738

[23] Neeland IJ, Poirier P, Després JP. Cardiovascular and Metabolic Heterogeneity of Obesity: Clinical Challenges and Implications for Management. Circulation. 2018;137(13):1391–406. 10.1161/CIRCULATIONAHA.117.029617. 29581366 10.1161/CIRCULATIONAHA.117.029617 PMC5875734

[24] Cai X, Song S, Hu J, Zhu Q, Shen D, Yang W, et al. Association of the trajectory of plasma aldosterone concentration with the risk of cardiovascular disease in patients with hypertension: a cohort study. Sci Rep. 2024;14(1):4906. 10.1038/s41598-024-54971-4. 38418472 10.1038/s41598-024-54971-4 PMC10902285

[25] Lemieux L. Causes, relationships and explanations: the power and limitations of observational longitudinal imaging studies. Curr Opin Neurol. 2008;21(4):391. 10.1097/WCO.0b013e3283056a50. 18607197 10.1097/WCO.0b013e3283056a50

[26] UK Biobank Pharma Proteomics Project (UKB-PPP). UKB – Olink Explore 3072 – FAQ (B0–B6) [Internet]. Oxford: UK Biobank; [cited 2026 Feb 4]. Available from: https://biobank.ndph.ox.ac.uk/ukb/ukb/docs/Olink-3072_B0-B6_FAQ.pdf

