# Supplementary material for: Associations of plasma level of soluble LDL receptor with cardiovascular events and mortality in a large prospective cohort study
Source: Lipids Health Dis. 2026 Apr 10;25:134. doi: 10.1186/s12944-026-02920-7 (PMC13188685; doi:10.1186/s12944-026-02920-7)
Supplement: Supplementary file 1 — Supplementary Material 1. [file 12944_2026_2920_MOESM1_ESM.docx]

**Table S1.** Number of Participants and Events Included in Each Cox Regression Model for CVD Outcomes

| **Model** | **N included** | **Events** | | | |
| --- | --- | --- | --- | --- | --- |
|  |  | **MI** | **HF** | **CVD Mortality** | **All-cause Mortality** |
| Model 1 | 47,518 | 1,275 | 1,638 | 761 | 4,355 |
| Model 2 | 47,282 | 1,271 | 1,630 | 761 | 4,332 |
| Model 3 | 28,610 | 748 | 943 | 433 | 2,475 |
| Model 4 | 28,509 | 746 | 938 | 430 | 2,448 |
| Model 4 + TG | 27,222 | 715 | 891 | 410 | 2,332 |
| Model 4 + LDL-C | 27,187 | 716 | 888 | 411 | 2,332 |
| Model 4 + HDL-C | 24,953 | 656 | 816 | 378 | 2,155 |
| Model 4 + TG, LDL-C | 27,171 | 715 | 888 | 410 | 2,331 |
| Model 4 + TG, LDL-C, HDL-C | 24,894 | 656 | 814 | 378 | 2,154 |
| Model 4 +ApoB | 27,098 | 714 | 881 | 407 | 2,318 |
| Model 4 +ApoA1 | 24,821 | 652 | 810 | 374 | 2,140 |
| Model 4 +ApoE | 24,110 | 617 | 792 | 345 | 2,050 |
| Model 4 +Lp(a) | 21,863 | 587 | 697 | 316 | 1,839 |
| Model 4 + TNFa | 27,489 | 722 | 895 | 418 | 2,351 |
| Model 4 + CRP | 27,170 | 716 | 889 | 410 | 2,328 |

*Abbreviations*: CVD, cardiovascular disease; TG, triglycerides; LDL-C, low-density lipoprotein cholesterol; HDL-C, high-density lipoprotein cholesterol; ApoB, apolipoprotein B; ApoA1, apolipoprotein A1; ApoE, apolipoprotein E; Lp(a), lipoprotein(a); TNFα, tumor necrosis factor-alpha; CRP, C-reactive protein; BMI, body mass index

Model 1: unadjusted; Model 2: adjusted for age, sex, and ethnicity; Model 3: additionally adjusted for education, smoking, drinking, and physical activity (MET), diabetes, and hypertension, and CVD medication use; Model 4: additionally adjusted for BMI; Additional models included further adjustments to the clinical model (Model 4), resulting in varying degrees of missingness across models. Sample sizes reflect complete-case analysis for each model.

**Table S2.** Sensitivity Analysis for Missingness-Driven Sample Loss (Income, Met Score)

| **Outcome** | **Model** | **Events/N** | **HR (95% CI)** |
| --- | --- | --- | --- |
| MI | Model 3 (primary) | 748/28,610 | 1.36 (1.21, 1.53) |
| MI | Model 3′ (-income, MET score) | 1,163/43,494 | 1.38 (1.26, 1.51) |
| MI | Model 4 (primary) | 746/28,509 | 1.32 (1.17, 1.50) |
| MI | Model 4′(-income, MET score) | 1,159/43,316 | 1.33 (1.20, 1.46) |
| HF | Model 3 (primary) | 943/28,610 | 0.98 (0.88, 1.09) |
| HF | Model 3′ (-income, MET score) | 1,494/43,494 | 1.02 (0.94, 1.11) |
| HF | Model 4 (primary) | 938/28,509 | 0.84 (0.75, 0.94) |
| HF | Model 4′(-income, MET score) | 1,485/43316 | 0.85 (0.78, 0.93) |
| CVD mortality | Model 3 (primary) | 433/28,610 | 0.82 (0.70, 0.96) |
| CVD mortality | Model 3′ (-income, MET score) | 695/43,494 | 0.88 (0.78, 1.00) |
| CVD mortality | Model 4 (primary) | 430/28,509 | 0.76 (0.64, 0.90) |
| CVD mortality | Model 4′(-income, MET score) | 689/43,316 | 0.78 (0.68, 0.87) |
| All-cause mortality | Model 3 (primary) | 2,475/28,610 | 0.95 (0.89, 1.01) |
| All-cause mortality | Model 3′ (-income, MET score) | 3,935/43,494 | 0.95 (0.90, 1.00) |
| All-cause mortality | Model 4 (primary) | 2,448/28,509 | 0.90 (0.84, 0.97) |
| All-cause mortality | Model 4′(-income, MET score) | 3,891/43316 | 0.87 (0.84, 0.84) |

*Abbreviations:* CI, confidence interval; CVD, cardiovascular disease; HF, heart failure; HR, hazard ratio; MET, metabolic equivalent of task; MI, myocardial infarction.

Primary Model 3 and Model 4 are defined in Table S1. Model 3′/4′ repeat the corresponding primary model excluding income and MET score to mitigate missingness-related sample loss. HRs are estimated per 1-SD.

**Table S3.** Positive predictive value bias analysis for MI and HF

| **Outcome** | **Model** | **Assumed PPV** | **Primary Cox HR (95% CI)** | **PPV-adjusted HR (median)** | **PPV-adjusted 95% interval** | **N sims** |
| --- | --- | --- | --- | --- | --- | --- |
| MI | Model 3 | 0.8 | 1.36 (1.21, 1.53) | 1.357 | 1.353-1.360 | 1,000 |
|  | Model 4 | 0.8 | 1.32 (1.17, 1.50) | 1.324 | 1.320-1.327 | 1,000 |
| HF | Model 3 | 0.7 | 0.98 (0.88, 1.09) | 0.981 | 0.979-0.984 | 1,000 |
|  | Model 4 | 0.7 | 0.84 (0.75, 0.94) | 0.845 | 0.843-0.848 | 1,000 |

*Abbreviations*: CI, confidence interval; HF, heart failure; HR, hazard ratio; MI, myocardial infarction; PPV, positive predictive value.

PPV-adjusted estimates were obtained via simulation-based correction for outcome misclassification assuming non-differential false positives (MI PPV=0.80; HF PPV=0.70). “PPV-adjusted 95% interval” indicates the 2.5^th^-97.5th percentiles across simulations. N sims denotes the number of simulations (1,000).

**Table S4.** Cox proportional hazards models excluding incident events occurring within 3 years after baseline

|  | per 1-SD | | | | | | | |
| --- | --- | --- | --- | --- | --- | --- | --- | --- |
|  | **Myocardial Infarction** | | **Heart Failure** | | **CVD mortality** | | **All-cause mortality** | |
|  | HR (95% CI) | p | HR (95% CI) | p | HR (95% CI) | p | HR (95% CI) | p |
| Model 1 | 1.40 (1.28, 1.54) | **<0.001** | 1.17 (1.08, 1.27) | **<0.001** | 0.95 (0.85, 1.07) | 0.410 | 1.05 (1.00, 1.10) | 0.052 |
| Model 2 | 1.36 (1.23, 1.50) | **<0.001** | 1.08 (0.99, 1.18) | 0.066 | 0.87 (0.77, 0.99) | **0.034** | 0.95 (0.90, 1.00) | 0.050 |
| Model 3 | 1.28 (1.13, 1.45) | **<0.001** | 0.97 (0.87, 1.09) | 0.618 | 0.79 (0.67, 0.93) | **0.006** | 0.93 (0.87, 1.00) | **0.036** |
| Model 4: Model 3 + BMI | 1.25 (1.10, 1.43) | **0.001** | 0.83 (0.73, 0.93) | **0.002** | 0.72 (0.61, 0.86) | **<0.001** | 0.88 (0.82, 0.94) | **<0.001** |
| Model 4 + TG | 1.22 (1.02, 1.44) | **0.025** | 0.79 (0.67, 0.92) | **0.003** | 0.66 (0.53, 0.83) | **<0.001** | 0.87 (0.79, 0.95) | **0.003** |
| Model 4 + LDL-C | 1.05 (0.90, 1.22) | 0.573 | 0.89 (0.78, 1.01) | 0.081 | 0.66 (0.54, 0.81) | **<0.001** | 0.91 (0.84, 0.99) | **0.024** |
| Model 4 + HDL-C | 1.15 (0.99, 1.33) | **0.069** | 0.84 (0.73, 0.96) | **0.010** | 0.74 (0.61, 0.91) | **0.003** | 0.88 (0.81, 0.95) | **0.002** |
| Model 4 + TG, LDL-C | 1.02 (0.85, 1.24) | 0.809 | 0.83 (0.71, 0.98) | **0.025** | 0.60 (0.47, 0.77) | **<0.001** | 0.89 (0.81, 0.98) | **0.021** |
| Model 4 + TG, LDL-C, HDL-C | 0.95 (0.78, 1.17) | 0.653 | 0.85 (0.72, 1.01) | 0.064 | 0.61 (0.47, 0.78) | **<0.001** | 0.89 (0.81, 0.99) | **0.033** |
| Model 4 +ApoB | 1.01 (0.87, 1.19) | 0.854 | 0.86 (0.76, 0.99) | **0.032** | 0.63 (0.51, 0.77) | **<0.001** | 0.89 (0.82, 0.96) | **0.005** |
| Model 4 +ApoA1 | 1.16 (1.01, 1.34) | 0.040 | 0.84 (0.74, 0.96) | **0.011** | 0.74 (0.61, 0.89) | **0.002** | 0.88 (0.82, 0.96) | **0.002** |
| Model 4 +ApoE | 1.26 (1.08, 1.46) | **0.003** | 0.81 (0.71, 0.93) | **0.002** | 0.71 (0.58, 0.87) | **0.001** | 0.89 (0.82, 0.97) | **0.007** |
| Model 4 +Lp(a) | 1.29 (1.11, 1.50) | **0.001** | 0.82 (0.71, 0.94) | **0.004** | 0.69 (0.56, 0.85) | **<0.001** | 0.89 (0.82, 0.97) | **0.006** |
| Model 4 + TNFa | 1.23 (1.08, 1.41) | **0.002** | 0.81 (0.72, 0.92) | **0.001** | 0.70 (0.58, 0.84) | **<0.001** | 0.85 (0.79, 0.92) | **<0.001** |
| Model 4 + CRP | 1.21 (1.06, 1.39) | **0.006** | 0.82 (0.72, 0.92) | **0.001** | 0.70 (0.59, 0.84) | **<0.001** | 0.87 (0.80, 0.93) | **<0.001** |

*Abbreviations*: CVD, cardiovascular disease; TG, triglycerides; LDL-C, low-density lipoprotein cholesterol; HDL-C, high-density lipoprotein cholesterol; ApoB, apolipoprotein B; ApoA1, apolipoprotein A1; ApoE, apolipoprotein E; Lp(a), lipoprotein(a); TNFα, tumor necrosis factor-alpha; CRP, C-reactive protein; BMI, body mass index

Model 1: unadjusted; Model 2: adjusted for age, sex, and ethnicity; Model 3: additionally adjusted for education, smoking, drinking, and physical activity (MET), diabetes, and hypertension, and CVD medication use; Model 4: additionally adjusted for BMI; Additional models included further adjustments to the clinical model (Model 4), resulting in varying degrees of missingness across models.

**Table S5.** Associations of sLDLR Tertiles with Clinical Outcomes

(A)

| **Acute Myocardial Infarction** | | | | | | | |
| --- | --- | --- | --- | --- | --- | --- | --- |
|  | **T1** | | **T2** | | | **T3** | |
| Case / Person years | 326 / 209,378.46 | | 419 / 208,077.06 | | | 530 / 207,612.69 | |
|  |  |  | | HR (95% CI) | p | HR (95% CI) | p |
| Model 1 | 1.00 (ref) | | 1.30 (1.12, 1.50) | | **<0.001** | 1.64 (1.43, 1.88) | **<0.001** |
| Model 2 | 1.00 (ref) | | 1.17 (1.01, 1.35) | | **0.037** | 1.53 (1.33, 1.76) | **<0.001** |
| Model 3 | 1.00 (ref) | | 1.19 (0.99, 1.44) | | 0.066 | 1.48 (1.23, 1.77) | **<0.001** |
| Model 4 | 1.00 (ref) | | 1.17 (0.97, 1.42) | | 0.108 | 1.42 (1.17, 1.71) | **<0.001** |
| Model 4 + TG | 1.00 (ref) | | 1.13 (0.93, 1.38) | | 0.217 | 1.29 (1.04, 1.62) | **0.024** |
| Model 4 + LDL-C | 1.00 (ref) | | 0.99 (0.81, 1.21) | | 0.923 | 1.03 (0.84, 1.28) | 0.749 |
| Model 4 + HDL-C | 1.00 (ref) | | 1.11 (0.91, 1.37) | | 0.299 | 1.27 (1.03, 1.56) | **0.024** |
| Model 4 + TG, LDL-C | 1.00 (ref) | | 0.98 (0.80, 1.20) | | 0.832 | 0.99 (0.78, 1.25) | 0.936 |
| Model 4 + TG, LDL-C, HDL-C | 1.00 (ref) | | 0.94 (0.76, 1.16) | | 0.545 | 0.93 (0.73, 1.19) | 0.577 |
| Model 4 +ApoB | 1.00 (ref) | | 0.97 (0.80, 1.18) | | 0.767 | 0.98 (0.80, 1.22) | 0.886 |
| Model 4 +ApoA1 | 1.00 (ref) | | 1.12 (0.92, 1.37) | | 0.267 | 1.29 (1.06, 1.58) | **0.013** |
| Model 4 +ApoE | 1.00 (ref) | | 1.18 (0.95, 1.45) | | 0.131 | 1.42 (1.15, 1.75) | **0.001** |
| Model 4 +Lp(a) | 1.00 (ref) | | 1.15 (0.93, 1.42) | | 0.208 | 1.42 (1.15, 1.75) | **0.001** |
| Model 4 + TNFa | 1.00 (ref) | | 1.13 (0.93, 1.37) | | 0.221 | 1.36 (1.12, 1.65) | **0.002** |
| Model 4 + CRP | 1.00 (ref) | | 1.14 (0.94, 1.39) | | 0.180 | 1.34 (1.10, 1.62) | **0.003** |

(B)

| **Heart Failure** | | | | | | | |
| --- | --- | --- | --- | --- | --- | --- | --- |
|  | **T1** | **T2** | | | | **T3** | |
| Case / Person years | 495 / 209,030.55 | 538 / 208,167.19 | | | | 605 / 208,249.99 | |
|  |  | |  | HR (95% CI) | p | HR (95% CI) | p |
| Model 1 | 1.00 (ref) | 1.09 (0.97, 1.24) | | | 0.149 | 1.23 (1.09, 1.38) | **0.001** |
| Model 2 | 1.00 (ref) | 0.96 (0.85, 1.09) | | | 0.516 | 1.10 (0.97, 1.24) | 0.125 |
| Model 3 | 1.00 (ref) | 1.01 (0.86, 1.18) | | | 0.934 | 0.98 (0.83, 1.15) | 0.771 |
| Model 4 | 1.00 (ref) | 0.90 (0.76, 1.05) | | | 0.181 | 0.79 (0.67, 0.94) | **0.007** |
| Model 4 + TG | 1.00 (ref) | 0.90 (0.76, 1.06) | | | 0.214 | 0.78 (0.64, 0.96) | **0.018** |
| Model 4 + LDL-C | 1.00 (ref) | 0.94 (0.79, 1.12) | | | 0.484 | 0.86 (0.71, 1.03) | 0.101 |
| Model 4 + HDL-C | 1.00 (ref) | 0.92 (0.77, 1.10) | | | 0.346 | 0.83 (0.69, 0.99) | **0.043** |
| Model 4 + TG, LDL-C | 1.00 (ref) | 0.93 (0.78, 1.10) | | | 0.384 | 0.82 (0.66, 1.01) | 0.063 |
| Model 4 + TG, LDL-C, HDL-C | 1.00 (ref) | 0.95 (0.79, 1.15) | | | 0.619 | 0.87 (0.70, 1.09) | 0.224 |
| Model 4 +ApoB | 1.00 (ref) | 0.93 (0.79, 1.11) | | | 0.438 | 0.83 (0.69, 1.00) | 0.051 |
| Model 4 +ApoA1 | 1.00 (ref) | 0.92 (0.77, 1.10) | | | 0.348 | 0.83 (0.69, 0.99) | **0.042** |
| Model 4 +ApoE | 1.00 (ref) | 0.91 (0.76, 1.08) | | | 0.265 | 0.76 (0.63, 0.91) | **0.003** |
| Model 4 +Lp(a) | 1.00 (ref) | 0.94 (0.78, 1.13) | | | 0.523 | 0.81 (0.67, 0.99) | **0.036** |
| Model 4 + TNFa | 1.00 (ref) | 0.88 (0.74, 1.03) | | | 0.116 | 0.76 (0.64, 0.90) | **0.001** |
| Model 4 + CRP | 1.00 (ref) | 0.91 (0.77, 1.07) | | | 0.252 | 0.79 (0.67, 0.94) | **0.007** |

(C)

| **CVD Mortality** | | | | | | | |
| --- | --- | --- | --- | --- | --- | --- | --- |
|  | **T1** | **T2** | | | | **T3** | |
| Case / Person years | 272 / 211,983.03 | 238 / 211,290 | | | | 251 / 211,467.37 | |
|  |  | |  | HR (95% CI) | p | HR (95% CI) | p |
| Model 1 | 1.00 (ref) | 0.88 (0.74, 1.05) | | | 0.153 | 0.93 (0.78, 1.10) | 0.377 |
| Model 2 | 1.00 (ref) | 0.78 (0.65, 0.92) | | | **0.004** | 0.84 (0.71, 1.00) | 0.055 |
| Model 3 | 1.00 (ref) | 0.78 (0.62, 0.98) | | | **0.034** | 0.70 (0.55, 0.88) | **0.002** |
| Model 4 | 1.00 (ref) | 0.74 (0.59, 0.93) | | | **0.010** | 0.62 (0.49, 0.80) | **<0.001** |
| Model 4 + TG | 1.00 (ref) | 0.68 (0.53, 0.87) | | | **0.002** | 0.55 (0.41, 0.74) | **<0.001** |
| Model 4 + LDL-C | 1.00 (ref) | 0.66 (0.52, 0.84) | | | **0.001** | 0.55 (0.42, 0.71) | **<0.001** |
| Model 4 + HDL-C | 1.00 (ref) | 0.71 (0.55, 0.91) | | | **0.007** | 0.65 (0.50, 0.84) | **0.001** |
| Model 4 + TG, LDL-C | 1.00 (ref) | 0.64 (0.50, 0.82) | | | **<0.001** | 0.49 (0.36, 0.67) | **<0.001** |
| Model 4 + TG, LDL-C, HDL-C | 1.00 (ref) | 0.63 (0.48, 0.81) | | | **<0.001** | 0.49 (0.36, 0.68) | **<0.001** |
| Model 4 +ApoB | 1.00 (ref) | 0.66 (0.52, 0.84) | | | **0.001** | 0.53 (0.41, 0.70) | **<0.001** |
| Model 4 +ApoA1 | 1.00 (ref) | 0.68 (0.53, 0.87) | | | **0.002** | 0.63 (0.49, 0.81) | **<0.001** |
| Model 4 +ApoE | 1.00 (ref) | 0.75 (0.58, 0.97) | | | **0.027** | 0.60 (0.46, 0.79) | **<0.001** |
| Model 4 +Lp(a) | 1.00 (ref) | 0.59 (0.45, 0.78) | | | **<0.001** | 0.56 (0.43, 0.74) | **<0.001** |
| Model 4 + TNFa | 1.00 (ref) | 0.72 (0.57, 0.92) | | | **0.007** | 0.60 (0.47, 0.76) | **<0.001** |
| Model 4 + CRP | 1.00 (ref) | 0.70 (0.55, 0.88) | | | **0.003** | 0.60 (0.47, 0.77) | **<0.001** |

(D)

| **All-cause Mortality** | | | | | | | |
| --- | --- | --- | --- | --- | --- | --- | --- |
|  | **T1** | **T2** | | | | **T3** | |
| Case / Person years | 1,388 / 211983.03 | 1,494 / 211290 | | | | 1,473 / 211467.37 | |
|  |  | |  | HR (95% CI) | p | HR (95% CI) | p |
| Model 1 | 1.00 (ref) | 1.08 (1.01, 1.16) | | | **0.035** | 1.06 (0.99, 1.14) | 0.097 |
| Model 2 | 1.00 (ref) | 0.94 (0.88, 1.01) | | | 0.113 | 0.94 (0.87, 1.01) | 0.088 |
| Model 3 | 1.00 (ref) | 0.96 (0.87, 1.05) | | | 0.365 | 0.92 (0.83, 1.01) | 0.087 |
| Model 4 | 1.00 (ref) | 0.93 (0.84, 1.02) | | | 0.127 | 0.86 (0.78, 0.95) | **0.004** |
| Model 4 + TG | 1.00 (ref) | 0.92 (0.82, 1.02) | | | 0.100 | 0.85 (0.75, 0.96) | **0.011** |
| Model 4 + LDL-C | 1.00 (ref) | 0.94 (0.85, 1.04) | | | 0.248 | 0.90 (0.81, 1.01) | 0.074 |
| Model 4 + HDL-C | 1.00 (ref) | 0.92 (0.83, 1.03) | | | 0.145 | 0.87 (0.77, 0.97) | **0.012** |
| Model 4 + TG, LDL-C | 1.00 (ref) | 0.93 (0.84, 1.04) | | | 0.206 | 0.88 (0.77, 1.00) | 0.056 |
| Model 4 + TG, LDL-C, HDL-C | 1.00 (ref) | 0.94 (0.84, 1.05) | | | 0.288 | 0.90 (0.78, 1.03) | 0.111 |
| Model 4 +ApoB | 1.00 (ref) | 0.93 (0.83, 1.03) | | | 0.149 | 0.87 (0.78, 0.98) | **0.017** |
| Model 4 +ApoA1 | 1.00 (ref) | 0.92 (0.83, 1.02) | | | 0.126 | 0.86 (0.77, 0.97) | **0.010** |
| Model 4 +ApoE | 1.00 (ref) | 0.93 (0.83, 1.04) | | | 0.188 | 0.87 (0.77, 0.97) | **0.013** |
| Model 4 +Lp(a) | 1.00 (ref) | 0.88 (0.78, 0.98) | | | **0.025** | 0.83 (0.74, 0.93) | **0.002** |
| Model 4 + TNFa | 1.00 (ref) | 0.93 (0.84, 1.02) | | | 0.134 | 0.83 (0.74, 0.92) | **<0.001** |
| Model 4 + CRP | 1.00 (ref) | 0.91 (0.82, 1.01) | | | 0.064 | 0.84 (0.75, 0.93) | **0.001** |

*Abbreviations*: sLDLR, soluble low-density lipoprotein receptor; CVD, Cardiovascular disease; TG, triglycerides; LDL-C, low-density lipoprotein cholesterol; HDL-C, high-density lipoprotein cholesterol; ApoB, apolipoprotein B; ApoA1, apolipoprotein A1; ApoE, apolipoprotein E; Lp(a), lipoprotein(a); TNFα, tumor necrosis factor-alpha; CRP, C-reactive protein; BMI, body mass index

T1, T2, and T3 indicate tertiles of sLDLR based on the number of participants. Model 1: unadjusted; Model 2: adjusted for age, sex, and ethnicity; Model 3: additionally adjusted for education, smoking, drinking, and physical activity (MET), diabetes, hypertension and CVD medication use; Model 4: additionally adjusted for BMI; Additional models included further adjustments to Model 4, resulting in varying degrees of missingness across models. Case / Person years were calculated based on Model 1. Sample sizes reflect complete-case analysis for each model.

**Table S6.** Number of Events and Non-events Used for Time Dependent ROC Analysis of Each Outcome at 5 and 10 Years

| Outcomes | **Follow-up Time** | **Valid Samples (N)** | **Events (N)** | **Non-events (N)** |
| --- | --- | --- | --- | --- |
| MI | 5 years | 8,407 | 58 | 8,349 |
|  | 10 years | 8,130 | 153 | 7,977 |
| HF | 5 years | 8,406 | 53 | 8,353 |
|  | 10 years | 8,167 | 165 | 8,002 |
| CVD death | 5 years | 8,421 | 20 | 8,401 |
|  | 10 years | 8,183 | 65 | 8,118 |
| All-cause death | 5 years | 8,539 | 133 | 8,406 |
|  | 10 years | 8,531 | 429 | 8,102 |

*Abbreviations*: ROC, receiver operating characteristic; CVD, cardiovascular disease; MI, myocardial Infarction; HF, heart failure.

Analyses were based on a training-test split, with 30% of the data used as the test set and reflected in the reported results.

**Table S7.** Baseline characteristics of the full UKB-PPP cohort and the analytic cohort

| var | Full mean | Full SD | M3 mean | M3 SD | SMD |
| --- | --- | --- | --- | --- | --- |
| Age | 56.805 | 8.212 | 56.002 | 8.191 | -0.098 |
| Male | 0.461 |  | 0.462 |  | 0.003 |
| Female | 0.539 |  | 0.538 |  | -0.003 |
| White | 0.937 |  | 0.945 |  | 0.031 |
| Non-white | 0.063 |  | 0.055 |  | -0.031 |
| Smoking history |  |  |  |  |  |
| Never | 0.543 |  | 0.554 |  | 0.022 |
| Past | 0.35 |  | 0.343 |  | -0.016 |
| Current | 0.106 |  | 0.103 |  | -0.011 |
| Alcohol drinking history |  |  |  |  |  |
| Never | 0.047 |  | 0.039 |  | -0.041 |
| Past | 0.039 |  | 0.035 |  | -0.022 |
| Current | 0.914 |  | 0.926 |  | 0.046 |
| MET score | 2621.487 | 2624.174 | 2619.606 | 2615.607 | -0.001 |
| DM (proportion) | 0.737 |  | 0.745 |  | 0.017 |
| HTN (proportion) | 0.33 |  | 0.342 |  | 0.024 |
| BMI, Kg/m2 | 27.477 | 4.802 | 27.307 | 4.7 | -0.036 |
| Triglycerides, mmol/L | 1.744 | 1.02 | 1.726 | 1.013 | -0.018 |
| LDL-C, mmol/L | 3.53 | 0.881 | 3.584 | 0.858 | 0.062 |
| HDL-C, mmol/L | 1.443 | 0.383 | 1.456 | 0.379 | 0.033 |
| ApoB, g/L | 1.026 | 0.241 | 1.037 | 0.238 | 0.046 |
| ApoA1, g/L | 1.534 | 0.272 | 1.541 | 0.268 | 0.025 |
| ApoE | 0.052 | 0.739 | 0.055 | 0.74 | 0.004 |
| Lp(a), nmol/L | 45.134 | 49.31 | 44.89 | 49.273 | -0.005 |
| TNF-α | 0.034 | 0.434 | 0.012 | 0.421 | -0.051 |
| CRP, mg/L | 2.658 | 4.444 | 2.535 | 4.299 | -0.028 |
| sLDLR | 0.009 | 0.628 | -0.005 | 0.627 | -0.023 |

*Abbreviations*: ApoA1, apolipoprotein A1; ApoB, apolipoprotein B; ApoE, apolipoprotein E; BMI, body mass index; CRP, C-reactive protein; Full, full UKB-PPP cohort; HDL-C, high-density lipoprotein cholesterol; LDL-C, low-density lipoprotein cholesterol; Lp(a), lipoprotein(a); M3, Model 3; sLDLR, soluble low-density lipoprotein receptor; SMD, standardized mean difference; TNF-α, tumor necrosis factor-alpha; UKB-PPP, UK Biobank Pharma Proteomics Project.

**Note**: Values are mean (SD) for continuous variables and proportion for categorical variables. SMD indicates standardized mean difference (analytic cohort minus full cohort); absolute values <0.10 were considered small, suggesting minimal differences between cohorts.

**Table S8**. Bonferroni- and FDR-adjusted P-values for associations between sLDLR and cardiovascular outcomes

| Outcome | Model | Exposure  (sLDLR) | contrast | HR (95% CI) | p | p_bonf | q |
| --- | --- | --- | --- | --- | --- | --- | --- |
| MI | M1 | cont |  | 1.43 (1.31, 1.56) | 0.000 | 0.000 | 0.000 |
| MI | M2 | cont |  | 1.39 (1.27, 1.52) | 0.000 | 0.000 | 0.000 |
| MI | M3 | cont |  | 1.36 (1.21, 1.53) | 0.000 | 0.000 | 0.000 |
| MI | M4 | cont |  | 1.32 (1.17, 1.50) | 0.000 | 0.001 | 0.000 |
| MI | M4+ApoA1 | cont |  | 1.23 (1.08, 1.40) | 0.002 | 0.430 | 0.008 |
| MI | M4+ApoB | cont |  | 1.06 (0.91, 1.22) | 0.448 | 1.000 | 0.500 |
| MI | M4+ApoE | cont |  | 1.33 (1.16, 1.53) | 0.000 | 0.009 | 0.001 |
| MI | M4+HDL | cont |  | 1.21 (1.06, 1.39) | 0.005 | 0.985 | 0.014 |
| MI | M4+LDL | cont |  | 1.08 (0.94, 1.25) | 0.265 | 1.000 | 0.317 |
| MI | M4+Lp(a) | cont |  | 1.38 (1.21, 1.59) | 0.000 | 0.001 | 0.000 |
| MI | M4+TG | cont |  | 1.28 (1.10, 1.50) | 0.002 | 0.277 | 0.006 |
| MI | M4+TG+LDL | cont |  | 1.06 (0.89, 1.26) | 0.493 | 1.000 | 0.517 |
| MI | M4+TG+LDL+HDL | cont |  | 0.99 (0.82, 1.20) | 0.949 | 1.000 | 0.954 |
| MI | M4+TNFa | cont |  | 1.30 (1.15, 1.48) | 0.000 | 0.006 | 0.000 |
| MI | M4+logCRP | cont |  | 1.28 (1.13, 1.45) | 0.000 | 0.025 | 0.001 |
| MI | M1 | tert | T2vsT1 | 1.30 (1.12, 1.50) | 0.000 | 0.094 | 0.003 |
| MI | M1 | tert | T3vsT1 | 1.64 (1.43, 1.88) | 0.000 | 0.000 | 0.000 |
| MI | M2 | tert | T2vsT1 | 1.17 (1.01, 1.35) | 0.370 | 1.000 | 0.071 |
| MI | M2 | tert | T3vsT1 | 1.53 (1.33, 1.76) | 0.000 | 0.000 | 0.000 |
| MI | M3 | tert | T2vsT1 | 1.19 (0.99, 1.44) | 0.066 | 1.000 | 0.115 |
| MI | M3 | tert | T3vsT1 | 1.48 (1.23, 1.77) | 0.000 | 0.005 | 0.000 |
| MI | M4 | tert | T2vsT1 | 1.17 (0.97, 1.42) | 0.108 | 1.000 | 0.164 |
| MI | M4 | tert | T3vsT1 | 1.42 (1.17, 1.71) | 0.000 | 0.054 | 0.002 |
| MI | M4+ApoA1 | tert | T2vsT1 | 1.12 (0.92, 1.37) | 0.267 | 1.000 | 0.316 |
| MI | M4+ApoA1 | tert | T3vsT1 | 1.29 (1.06, 1.58) | 0.013 | 1.000 | 0.027 |
| MI | M4+ApoB | tert | T2vsT1 | 0.97 (0.80, 1.18) | 0.767 | 1.000 | 0.766 |
| MI | M4+ApoB | tert | T3vsT1 | 0.98 (0.80, 1.22) | 0.886 | 1.000 | 0.897 |
| MI | M4+ApoE | tert | T2vsT1 | 1.18 (0.95, 1.45) | 0.131 | 1.000 | 0.176 |
| MI | M4+ApoE | tert | T3vsT1 | 1.42 (1.15, 1.75) | 0.001 | 0.201 | 0.005 |
| MI | M4+HDL | tert | T2vsT1 | 1.11 (0.91, 1.37) | 0.299 | 1.000 | 0.347 |
| MI | M4+HDL | tert | T3vsT1 | 1.27 (1.03, 1.56) | 0.024 | 1.000 | 0.044 |
| MI | M4+LDL | tert | T2vsT1 | 0.99 (0.81, 1.21) | 0.923 | 1.000 | 0.897 |
| MI | M4+LDL | tert | T3vsT1 | 1.03 (0.84, 1.28) | 0.749 | 1.000 | 0.791 |
| MI | M4+Lp(a) | tert | T2vsT1 | 1.15 (0.93, 1.42) | 0.208 | 1.000 | 0.281 |
| MI | M4+Lp(a) | tert | T3vsT1 | 1.42 (1.15, 1.75) | 0.001 | 0.223 | 0.005 |
| MI | M4+TG | tert | T2vsT1 | 1.13 (0.93, 1.38) | 0.217 | 1.000 | 0.281 |
| MI | M4+TG | tert | T3vsT1 | 1.29 (1.04, 1.62) | 0.024 | 1.000 | 0.041 |
| MI | M4+TG+LDL | tert | T2vsT1 | 0.98 (0.80, 1.20) | 0.832 | 1.000 | 0.828 |
| MI | M4+TG+LDL | tert | T3vsT1 | 0.99 (0.78, 1.25) | 0.936 | 1.000 | 0.957 |
| MI | M4+TG+LDL+HDL | tert | T2vsT1 | 0.94 (0.76, 1.16) | 0.545 | 1.000 | 0.588 |
| MI | M4+TG+LDL+HDL | tert | T3vsT1 | 0.93 (0.73, 1.19) | 0.577 | 1.000 | 0.618 |
| MI | M4+TNFa | tert | T2vsT1 | 1.13 (0.93, 1.37) | 0.221 | 1.000 | 0.292 |
| MI | M4+TNFa | tert | T3vsT1 | 1.36 (1.12, 1.65) | 0.002 | 0.306 | 0.006 |
| MI | M4+logCRP | tert | T2vsT1 | 1.14 (0.94, 1.39) | 0.180 | 1.000 | 0.248 |
| MI | M4+logCRP | tert | T3vsT1 | 1.34 (1.10, 1.62) | 0.003 | 0.580 | 0.009 |
| HF | M1 | cont |  | 1.16 (1.07, 1.25) | 0.000 | 0.036 | 0.001 |
| HF | M2 | cont |  | 1.07 (0.99, 1.16) | 0.103 | 1.000 | 0.148 |
| HF | M3 | cont |  | 0.98 (0.88, 1.09) | 0.714 | 1.000 | 0.751 |
| HF | M4 | cont |  | 0.84 (0.75, 0.94) | 0.003 | 0.580 | 0.009 |
| HF | M4+ApoA1 | cont |  | 0.85 (0.76, 0.97) | 0.013 | 1.000 | 0.027 |
| HF | M4+ApoB | cont |  | 0.86 (0.76, 0.98) | 0.019 | 1.000 | 0.037 |
| HF | M4+ApoE | cont |  | 0.82 (0.73, 0.93) | 0.003 | 0.451 | 0.008 |
| HF | M4+HDL | cont |  | 0.85 (0.75, 0.96) | 0.012 | 1.000 | 0.026 |
| HF | M4+LDL | cont |  | 0.88 (0.78, 1.00) | 0.050 | 1.000 | 0.084 |
| HF | M4+Lp(a) | cont |  | 0.84 (0.74, 0.96) | 0.012 | 1.000 | 0.026 |
| HF | M4+TG | cont |  | 0.81 (0.70, 0.93) | 0.004 | 0.760 | 0.011 |
| HF | M4+TG+LDL | cont |  | 0.83 (0.71, 0.97) | 0.019 | 1.000 | 0.037 |
| HF | M4+TG+LDL+HDL | cont |  | 0.86 (0.73, 1.01) | 0.062 | 1.000 | 0.100 |
| HF | M4+TNFa | cont |  | 0.83 (0.74, 0.93) | 0.002 | 0.316 | 0.006 |
| HF | M4+logCRP | cont |  | 0.83 (0.74, 0.94) | 0.002 | 0.399 | 0.007 |
| HF | M1 | tert | T2vsT1 | 1.09 (0.97, 1.24) | 0.149 | 1.000 | 0.194 |
| HF | M1 | tert | T3vsT1 | 1.23 (1.09, 1.38) | 0.001 | 0.124 | 0.003 |
| HF | M2 | tert | T2vsT1 | 0.96 (0.85, 1.09) | 0.516 | 1.000 | 0.563 |
| HF | M2 | tert | T3vsT1 | 1.10 (0.97, 1.24) | 0.125 | 1.000 | 0.171 |
| HF | M3 | tert | T2vsT1 | 1.01 (0.86, 1.18) | 0.934 | 1.000 | 0.945 |
| HF | M3 | tert | T3vsT1 | 0.98 (0.83, 1.15) | 0.771 | 1.000 | 0.797 |
| HF | M4 | tert | T2vsT1 | 0.90 (0.76, 1.05) | 0.181 | 1.000 | 0.233 |
| HF | M4 | tert | T3vsT1 | 0.79 (0.67, 0.94) | 0.007 | 1.000 | 0.017 |
| HF | M4+ApoA1 | tert | T2vsT1 | 0.92 (0.77, 1.10) | 0.348 | 1.000 | 0.399 |
| HF | M4+ApoA1 | tert | T3vsT1 | 0.83 (0.69, 0.99) | 0.042 | 1.000 | 0.072 |
| HF | M4+ApoB | tert | T2vsT1 | 0.93 (0.79, 1.11) | 0.438 | 1.000 | 0.489 |
| HF | M4+ApoB | tert | T3vsT1 | 0.83 (0.69, 1.00) | 0.051 | 1.000 | 0.086 |
| HF | M4+ApoE | tert | T2vsT1 | 0.91 (0.76, 1.08) | 0.265 | 1.000 | 0.316 |
| HF | M4+ApoE | tert | T3vsT1 | 0.76 (0.63, 0.91) | 0.003 | 0.592 | 0.009 |
| HF | M4+HDL | tert | T2vsT1 | 0.92 (0.77, 1.10) | 0.346 | 1.000 | 0.399 |
| HF | M4+HDL | tert | T3vsT1 | 0.83 (0.69, 0.99) | 0.043 | 1.000 | 0.073 |
| HF | M4+LDL | tert | T2vsT1 | 0.94 (0.79, 1.12) | 0.484 | 1.000 | 0.532 |
| HF | M4+LDL | tert | T3vsT1 | 0.86 (0.71, 1.03) | 0.101 | 1.000 | 0.148 |
| HF | M4+Lp(a) | tert | T2vsT1 | 0.94 (0.78, 1.13) | 0.523 | 1.000 | 0.567 |
| HF | M4+Lp(a) | tert | T3vsT1 | 0.81 (0.67, 0.99) | 0.036 | 1.000 | 0.063 |
| HF | M4+TG | tert | T2vsT1 | 0.90 (0.76, 1.06) | 0.214 | 1.000 | 0.267 |
| HF | M4+TG | tert | T3vsT1 | 0.78 (0.64, 0.96) | 0.018 | 1.000 | 0.035 |
| HF | M4+TG+LDL | tert | T2vsT1 | 0.93 (0.78, 1.10) | 0.384 | 1.000 | 0.432 |
| HF | M4+TG+LDL | tert | T3vsT1 | 0.82 (0.66, 1.01) | 0.063 | 1.000 | 0.100 |
| HF | M4+TG+LDL+HDL | tert | T2vsT1 | 0.95 (0.79, 1.15) | 0.619 | 1.000 | 0.660 |
| HF | M4+TG+LDL+HDL | tert | T3vsT1 | 0.87 (0.70, 1.09) | 0.224 | 1.000 | 0.277 |
| HF | M4+TNF | tert | T2vsT1 | 0.88 (0.74, 1.03) | 0.116 | 1.000 | 0.162 |
| HF | M4+TNF | tert | T3vsT1 | 0.76 (0.64, 0.90) | 0.001 | 0.237 | 0.005 |
| HF | M4+logCRP | tert | T2vsT1 | 0.91 (0.77, 1.07) | 0.252 | 1.000 | 0.302 |
| HF | M4+logCRP | tert | T3vsT1 | 0.79 (0.67, 0.94) | 0.007 | 1.000 | 0.017 |
| CVD Mortality | M1 | cont |  | 0.98 (0.87, 1.09) | 0.680 | 1.000 | 0.720 |
| CVD Mortality | M2 | cont |  | 0.90 (0.80, 1.02) | 0.090 | 1.000 | 0.135 |
| CVD Mortality | M3 | cont |  | 0.82 (0.70, 0.96) | 0.016 | 1.000 | 0.033 |
| CVD Mortality | M4 | cont |  | 0.76 (0.64, 0.90) | 0.001 | 0.218 | 0.005 |
| CVD Mortality | M4+ApoA1 | cont |  | 0.76 (0.63, 0.92) | 0.004 | 0.653 | 0.010 |
| CVD Mortality | M4+ApoB | cont |  | 0.67 (0.55, 0.81) | 0.000 | 0.010 | 0.001 |
| CVD Mortality | M4+ApoE | cont |  | 0.73 (0.61, 0.89) | 0.002 | 0.284 | 0.006 |
| CVD Mortality | M4+HDL | cont |  | 0.77 (0.64, 0.93) | 0.007 | 1.000 | 0.017 |
| CVD Mortality | M4+LDL | cont |  | 0.68 (0.56, 0.83) | 0.000 | 0.020 | 0.001 |
| CVD Mortality | M4+Lp(a) | cont |  | 0.72 (0.59, 0.88) | 0.001 | 0.212 | 0.005 |
| CVD Mortality | M4+TG | cont |  | 0.67 (0.54, 0.83) | 0.000 | 0.051 | 0.002 |
| CVD Mortality | M4+TG+LDL | cont |  | 0.61 (0.48, 0.77) | 0.000 | 0.005 | 0.000 |
| CVD Mortality | M4+TG+LDL+HDL | cont |  | 0.61 (0.48, 0.78) | 0.000 | 0.013 | 0.001 |
| CVD Mortality | M4+TNFa | cont |  | 0.73 (0.62, 0.87) | 0.000 | 0.074 | 0.002 |
| CVD Mortality | M4+logCRP | cont |  | 0.73 (0.61, 0.87) | 0.000 | 0.069 | 0.002 |
| CVD Mortality | M1 | tert | T2vsT1 | 0.88 (0.74, 1.05) | 0.153 | 1.000 | 0.198 |
| CVD Mortality | M1 | tert | T3vsT1 | 0.93 (0.78, 1.10) | 0.377 | 1.000 | 0.427 |
| CVD Mortality | M2 | tert | T2vsT1 | 0.78 (0.65, 0.92) | 0.004 | 0.792 | 0.012 |
| CVD Mortality | M2 | tert | T3vsT1 | 0.84 (0.71, 1.00) | 0.055 | 1.000 | 0.091 |
| CVD Mortality | M3 | tert | T2vsT1 | 0.78 (0.62, 0.98) | 0.034 | 1.000 | 0.062 |
| CVD Mortality | M3 | tert | T3vsT1 | 0.70 (0.55, 0.88) | 0.002 | 0.424 | 0.008 |
| CVD Mortality | M4 | tert | T2vsT1 | 0.74 (0.59, 0.93) | 0.010 | 1.000 | 0.024 |
| CVD Mortality | M4 | tert | T3vsT1 | 0.62 (0.49, 0.80) | 0.000 | 0.025 | 0.001 |
| CVD Mortality | M4+ApoA1 | tert | T2vsT1 | 0.68 (0.53, 0.87) | 0.002 | 0.449 | 0.008 |
| CVD Mortality | M4+ApoA1 | tert | T3vsT1 | 0.63 (0.49, 0.81) | 0.000 | 0.079 | 0.002 |
| CVD Mortality | M4+ApoB | tert | T2vsT1 | 0.66 (0.52, 0.84) | 0.001 | 0.157 | 0.004 |
| CVD Mortality | M4+ApoB | tert | T3vsT1 | 0.53 (0.41, 0.70) | 0.000 | 0.001 | 0.000 |
| CVD Mortality | M4+ApoE | tert | T2vsT1 | 0.75 (0.58, 0.97) | 0.027 | 1.000 | 0.049 |
| CVD Mortality | M4+ApoE | tert | T3vsT1 | 0.60 (0.46, 0.79) | 0.000 | 0.054 | 0.002 |
| CVD Mortality | M4+HDL | tert | T2vsT1 | 0.71 (0.55, 0.91) | 0.007 | 1.000 | 0.017 |
| CVD Mortality | M4+HDL | tert | T3vsT1 | 0.65 (0.50, 0.84) | 0.001 | 0.223 | 0.005 |
| CVD Mortality | M4+LDL | tert | T2vsT1 | 0.66 (0.52, 0.84) | 0.001 | 0.162 | 0.004 |
| CVD Mortality | M4+LDL | tert | T3vsT1 | 0.55 (0.42, 0.71) | 0.000 | 0.002 | 0.000 |
| CVD Mortality | M4+Lp(a) | tert | T2vsT1 | 0.59 (0.45, 0.78) | 0.000 | 0.032 | 0.001 |
| CVD Mortality | M4+Lp(a) | tert | T3vsT1 | 0.56 (0.43, 0.74) | 0.000 | 0.008 | 0.001 |
| CVD Mortality | M4+TG | tert | T2vsT1 | 0.68 (0.53, 0.87) | 0.002 | 0.373 | 0.007 |
| CVD Mortality | M4+TG | tert | T3vsT1 | 0.55 (0.41, 0.74) | 0.000 | 0.012 | 0.001 |
| CVD Mortality | M4+TG+LDL | tert | T2vsT1 | 0.64 (0.50, 0.82) | 0.000 | 0.076 | 0.002 |
| CVD Mortality | M4+TG+LDL | tert | T3vsT1 | 0.49 (0.36, 0.67) | 0.000 | 0.001 | 0.000 |
| CVD Mortality | M4+TG+LDL+HDL | tert | T2vsT1 | 0.63 (0.48, 0.81) | 0.000 | 0.087 | 0.002 |
| CVD Mortality | M4+TG+LDL+HDL | tert | T3vsT1 | 0.49 (0.36, 0.68) | 0.000 | 0.003 | 0.000 |
| CVD Mortality | M4+TNF | tert | T2vsT1 | 0.72 (0.57, 0.92) | 0.007 | 1.000 | 0.017 |
| CVD Mortality | M4+TNF | tert | T3vsT1 | 0.60 (0.47, 0.76) | 0.000 | 0.007 | 0.000 |
| CVD Mortality | M4+logCRP | tert | T2vsT1 | 0.70 (0.55, 0.88) | 0.003 | 0.482 | 0.008 |
| CVD Mortality | M4+logCRP | tert | T3vsT1 | 0.60 (0.47, 0.77) | 0.000 | 0.009 | 0.001 |
| All cause Mortality | M1 | cont |  | 1.06 (1.01, 1.11) | 0.018 | 1.000 | 0.035 |
| All cause Mortality | M2 | cont |  | 0.96 (0.91, 1.01) | 0.110 | 1.000 | 0.157 |
| All cause Mortality | M3 | cont |  | 0.95 (0.89, 1.01) | 0.102 | 1.000 | 0.148 |
| All cause Mortality | M4 | cont |  | 0.90 (0.84, 0.97) | 0.004 | 0.677 | 0.010 |
| All cause Mortality | M4+ApoA1 | cont |  | 0.90 (0.84, 0.97) | 0.008 | 1.000 | 0.020 |
| All cause Mortality | M4+ApoB | cont |  | 0.91 (0.85, 0.99) | 0.025 | 1.000 | 0.046 |
| All cause Mortality | M4+ApoE | cont |  | 0.91 (0.84, 0.99) | 0.019 | 1.000 | 0.037 |
| All cause Mortality | M4+HDL | cont |  | 0.90 (0.83, 0.97) | 0.006 | 1.000 | 0.016 |
| All cause Mortality | M4+LDL | cont |  | 0.94 (0.87, 1.01) | 0.089 | 1.000 | 0.135 |
| All cause Mortality | M4+Lp(a) | cont |  | 0.90 (0.83, 0.98) | 0.014 | 1.000 | 0.028 |
| All cause Mortality | M4+TG | cont |  | 0.89 (0.81, 0.97) | 0.010 | 1.000 | 0.024 |
| All cause Mortality | M4+TG+LDL | cont |  | 0.91 (0.83, 1.00) | 0.054 | 1.000 | 0.089 |
| All cause Mortality | M4+TG+LDL+HDL | cont |  | 0.91 (0.83, 1.00) | 0.062 | 1.000 | 0.100 |
| All cause Mortality | M4+TNFa | cont |  | 0.87 (0.81, 0.94) | 0.000 | 0.032 | 0.001 |
| All cause Mortality | M4+logCRP | cont |  | 0.89 (0.83, 0.95) | 0.001 | 0.196 | 0.005 |
| All cause Mortality | M1 | tert | T2vsT1 | 1.08 (1.01, 1.16) | 0.035 | 1.000 | 0.062 |
| All cause Mortality | M1 | tert | T3vsT1 | 1.06 (0.99, 1.14) | 0.097 | 1.000 | 0.145 |
| All cause Mortality | M2 | tert | T2vsT1 | 0.94 (0.88, 1.01) | 0.113 | 1.000 | 0.158 |
| All cause Mortality | M2 | tert | T3vsT1 | 0.94 (0.87, 1.01) | 0.088 | 1.000 | 0.135 |
| All cause Mortality | M3 | tert | T2vsT1 | 0.96 (0.87, 1.05) | 0.365 | 1.000 | 0.416 |
| All cause Mortality | M3 | tert | T3vsT1 | 0.92 (0.83, 1.01) | 0.087 | 1.000 | 0.134 |
| All cause Mortality | M4 | tert | T2vsT1 | 0.93 (0.84, 1.02) | 0.127 | 1.000 | 0.172 |
| All cause Mortality | M4 | tert | T3vsT1 | 0.86 (0.78, 0.95) | 0.004 | 0.732 | 0.011 |
| All cause Mortality | M4+ApoA1 | tert | T2vsT1 | 0.92 (0.83, 1.02) | 0.126 | 1.000 | 0.171 |
| All cause Mortality | M4+ApoA1 | tert | T3vsT1 | 0.86 (0.77, 0.97) | 0.010 | 1.000 | 0.023 |
| All cause Mortality | M4+ApoB | tert | T2vsT1 | 0.93 (0.83, 1.03) | 0.149 | 1.000 | 0.194 |
| All cause Mortality | M4+ApoB | tert | T3vsT1 | 0.87 (0.78, 0.98) | 0.017 | 1.000 | 0.034 |
| All cause Mortality | M4+ApoE | tert | T2vsT1 | 0.93 (0.83, 1.04) | 0.188 | 1.000 | 0.241 |
| All cause Mortality | M4+ApoE | tert | T3vsT1 | 0.87 (0.77, 0.97) | 0.013 | 1.000 | 0.027 |
| All cause Mortality | M4+HDL | tert | T2vsT1 | 0.92 (0.83, 1.03) | 0.145 | 1.000 | 0.192 |
| All cause Mortality | M4+HDL | tert | T3vsT1 | 0.87 (0.77, 0.97) | 0.012 | 1.000 | 0.026 |
| All cause Mortality | M4+LDL | tert | T2vsT1 | 0.94 (0.85, 1.04) | 0.248 | 1.000 | 0.300 |
| All cause Mortality | M4+LDL | tert | T3vsT1 | 0.90 (0.81, 1.01) | 0.074 | 1.000 | 0.115 |
| All cause Mortality | M4+Lp(a) | tert | T2vsT1 | 0.88 (0.78, 0.98) | 0.025 | 1.000 | 0.046 |
| All cause Mortality | M4+Lp(a) | tert | T3vsT1 | 0.83 (0.74, 0.93) | 0.002 | 0.369 | 0.007 |
| All cause Mortality | M4+TG | tert | T2vsT1 | 0.92 (0.82, 1.02) | 0.100 | 1.000 | 0.147 |
| All cause Mortality | M4+TG | tert | T3vsT1 | 0.85 (0.75, 0.96) | 0.011 | 1.000 | 0.025 |
| All cause Mortality | M4+TG+LDL | tert | T2vsT1 | 0.93 (0.84, 1.04) | 0.206 | 1.000 | 0.260 |
| All cause Mortality | M4+TG+LDL | tert | T3vsT1 | 0.88 (0.77, 1.00) | 0.056 | 1.000 | 0.091 |
| All cause Mortality | M4+TG+LDL+HDL | tert | T2vsT1 | 0.94 (0.84, 1.05) | 0.288 | 1.000 | 0.336 |
| All cause Mortality | M4+TG+LDL+HDL | tert | T3vsT1 | 0.90 (0.78, 1.03) | 0.111 | 1.000 | 0.157 |
| All cause Mortality | M4+TNF | tert | T2vsT1 | 0.93 (0.84, 1.02) | 0.134 | 1.000 | 0.178 |
| All cause Mortality | M4+TNF | tert | T3vsT1 | 0.83 (0.74, 0.92) | 0.000 | 0.072 | 0.002 |
| All cause Mortality | M4+logCRP | tert | T2vsT1 | 0.91 (0.82, 1.01) | 0.064 | 1.000 | 0.101 |
| All cause Mortality | M4+logCRP | tert | T3vsT1 | 0.84 (0.75, 0.93) | 0.001 | 0.178 | 0.004 |

*Abbreviations:* ApoA1, apolipoprotein A1; ApoB, apolipoprotein B; ApoE, apolipoprotein E; BMI, body mass index; CI, confidence interval; cont, continuous; CRP, C-reactive protein; FDR, false discovery rate; HDL-C, high-density lipoprotein cholesterol; HR, hazard ratio; LDL-C, low-density lipoprotein cholesterol; Lp(a), lipoprotein(a); p, p-value; p-bonf, Bonferroni-adjusted p-value; q, FDR-adjusted p-value (q-value); sLDLR, soluble low-density lipoprotein receptor; tert, tertile; TNF-α, tumor necrosis factor-alpha

M1: unadjusted; M2: adjusted for age, sex, and ethnicity; M3: additionally adjusted for education, smoking, drinking, and physical activity (MET), diabetes, and hypertension, and CVD medication use; M4: additionally adjusted for BMI; T1, T2, and T3 indicate tertiles of sLDLR based on the number of participants.

**Note:** To account for multiple testing, Bonferroni correction and FDR adjustment were applied across the association models; p-bonf and FDR q-values (q) are reported.
