# Supplementary material for: Associations of plasma level of soluble LDL receptor with cardiovascular events and mortality in a large prospective cohort study
Source: Lipids Health Dis. 2026 Apr 10;25:134. doi: 10.1186/s12944-026-02920-7 (PMC13188685; doi:10.1186/s12944-026-02920-7)
Supplement: Supplementary file 2 — Supplementary Material 2. [file 12944_2026_2920_MOESM2_ESM.docx]

**Figure S1.** UKB Study Population Flowchart

UKB participants
(n= 502,370)

UKB participants with sLDLR
at baseline
(n=51,644)

Excluded (n = 4,126)

Participants with a baseline diagnosis of total ischemic heart disease, heart failure, stroke, Alzheimer’s disease, Parkinson’s disease, or vascular dementia

Final study population

(n=47,518)

MI cases = 1,275

HF cases = 1,638

CVD death cases = 761

All-cause death cases = 4,355

*Abbreviations*: sLDLR, soluble low-density lipoprotein receptor; MI, myocardial infarction; HF, Heart Failure; CVD, Cardiovascular Disease

Sample size varied slightly across models due to missing values in covariates (see Table S1 for details)

**Figure S2.** Directed acyclic graph (DAG) for the assumed relationships among sLDLR, lipids, BMI, and clinical outcomes

*
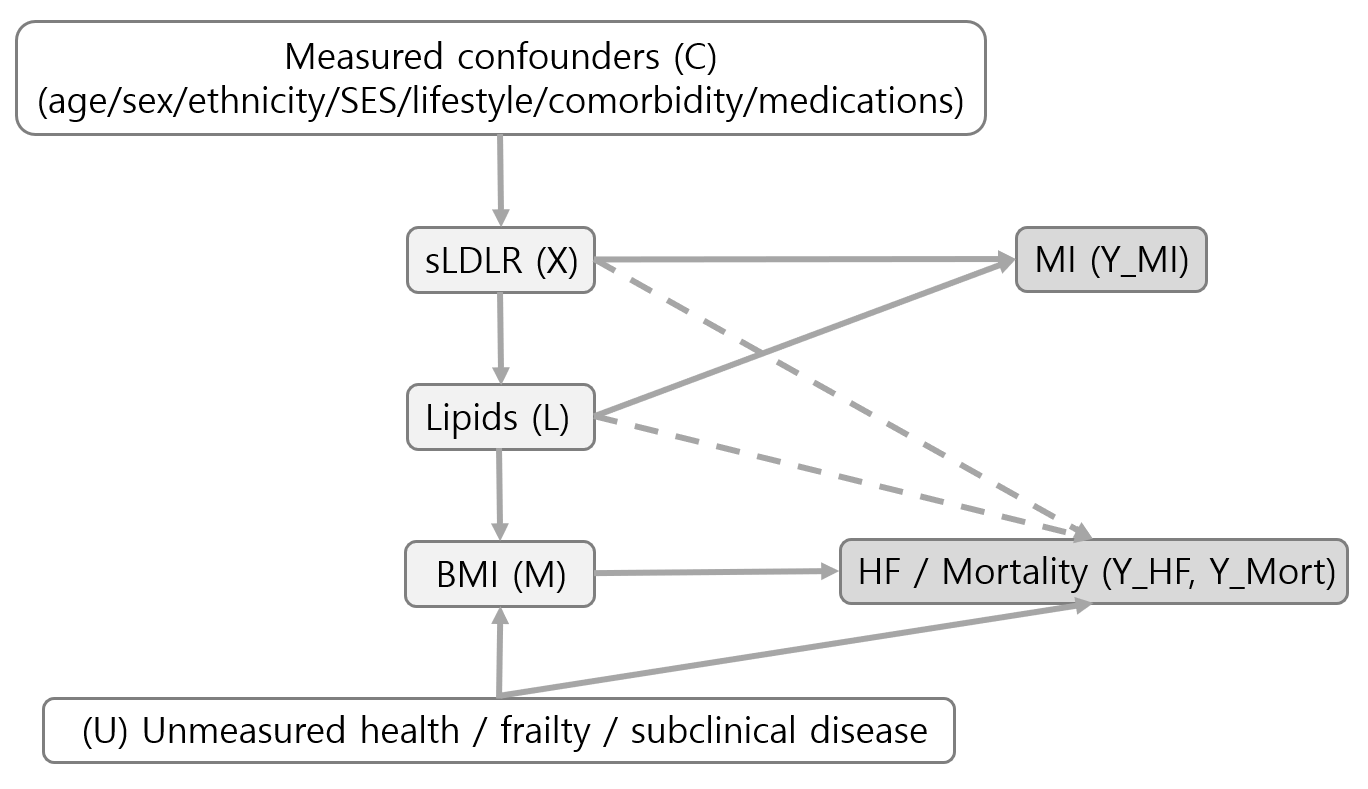
*

*Abbreviations*: BMI, body mass index; DAG, directed acyclic graph; HF, heart failure; L, lipids; MI, myocardial infarction; M, BMI; SES, socioeconomic status; sLDLR, soluble LDL receptor; U, unmeasured health status/subclinical disease; X, sLDLR; Y_MI, MI; Y_HF, HF; Y_Mort, mortality.

Note: BMI may be downstream of sLDLR-related lipid metabolism and may also reflect unmeasured health status (U); thus BMI-adjusted estimates are interpreted as BMI-conditional associations (not definitive causal effects). Dashed arrows indicate plausible but uncertain pathways.

**
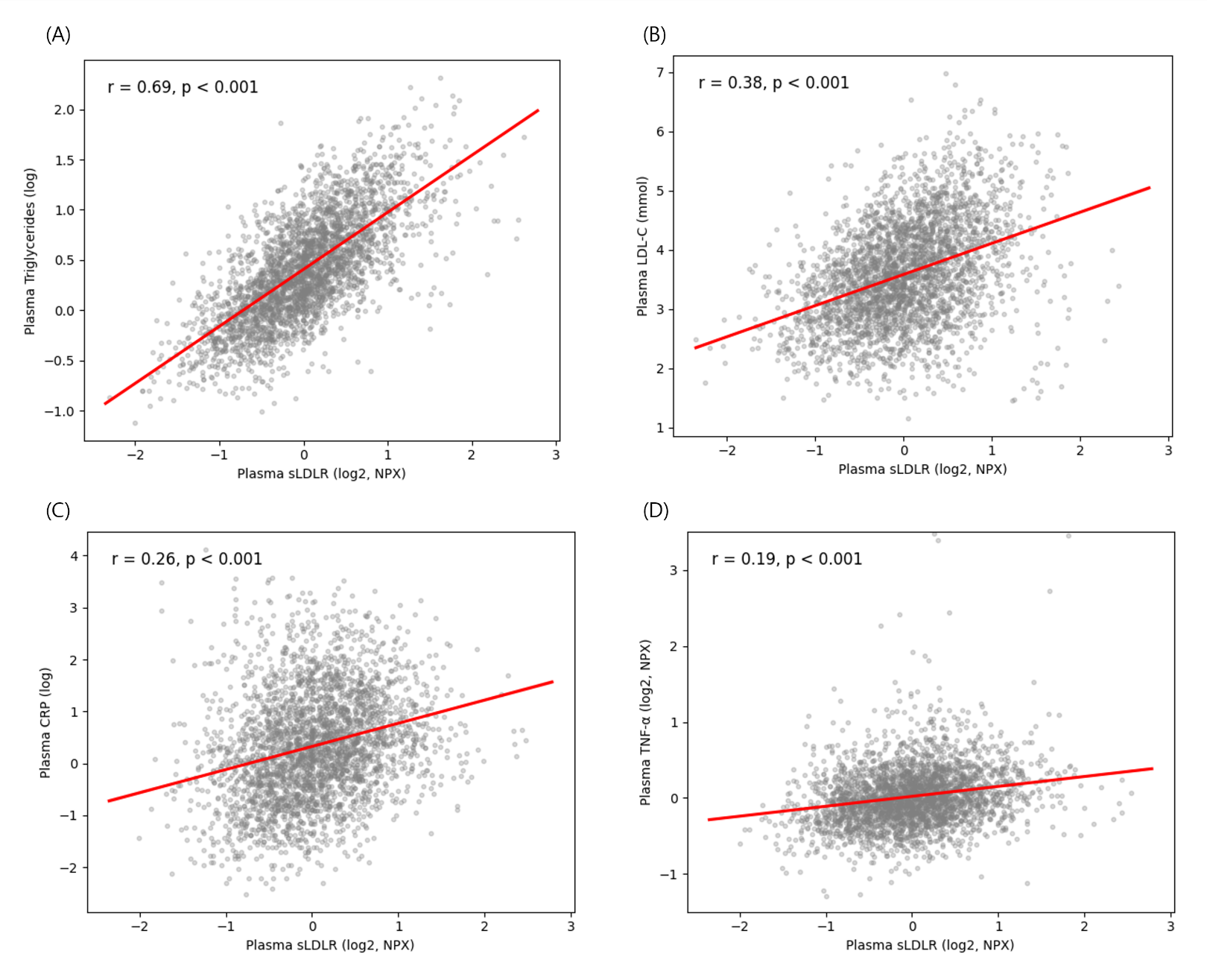
Figure S3.** Correlations Between Plasma sLDLR and Lipid and Inflammatory Biomarkers

*Abbreviations*: sLDLR, soluble low-density lipoprotein receptor; LDL-C, low-density lipoprotein cholesterol; CRP, C-reactive protein; TNFα, tumor necrosis factor-alpha.

Pearson correlation analysis between plasma soluble low-density lipoprotein receptor (sLDLR, log₂ NPX) and lipid or inflammatory biomarkers. (A) triglycerides (log-transformed), (B) LDL-C, (C) CRP (log-transformed), and (D) TNF-α (log₂ NPX). Red lines indicate linear regression fits. To improve visual clarity, scatter plots display a random subsample of 3,000 UKB participants per panel.

**Figure S4.** Subgroup Analyses of sLDLR With Clinical Outcomes Across BMI Tertiles
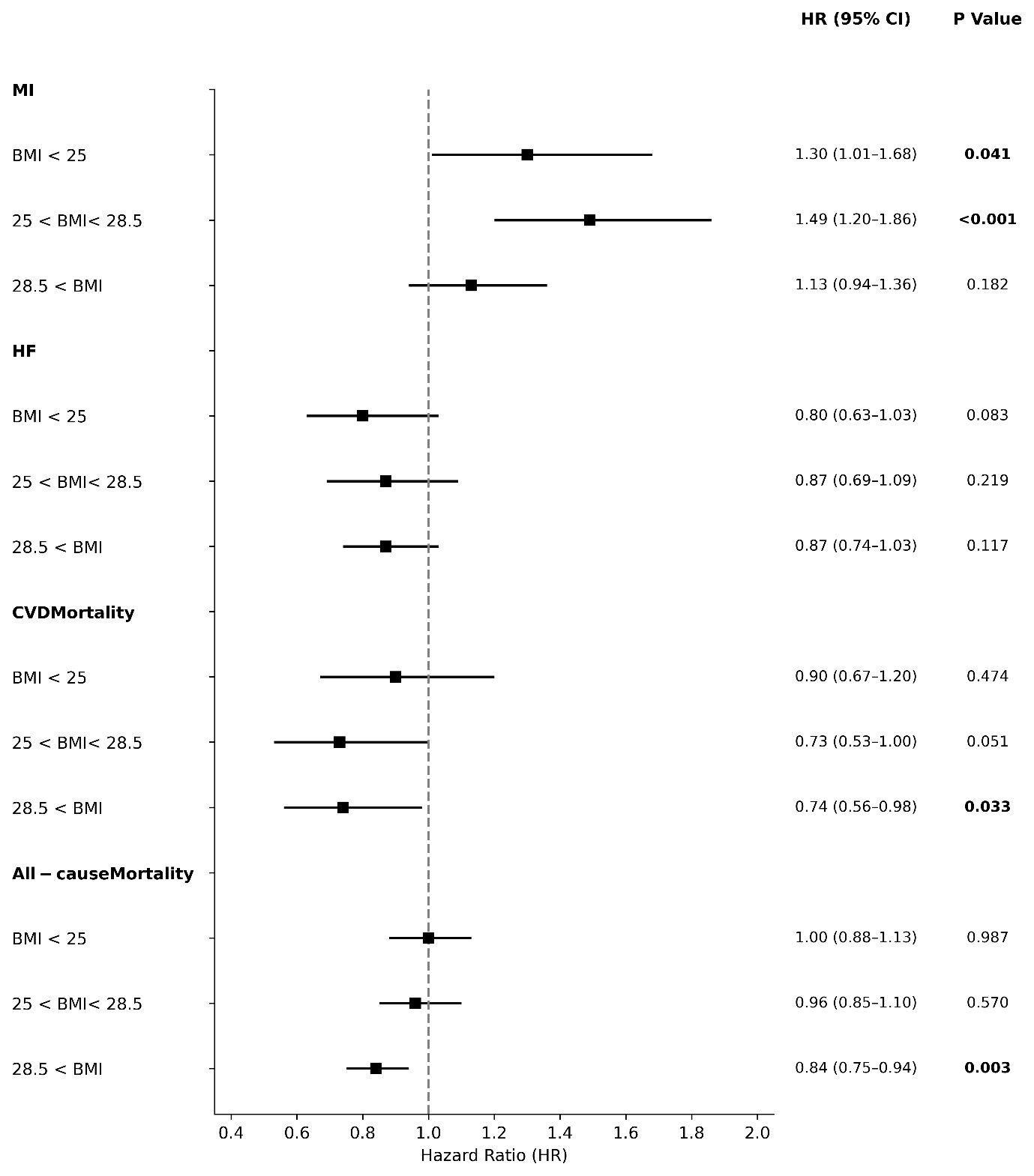


*Abbreviations*: sLDLR, soluble low-density lipoprotein receptor; BMI, body mass index; MI, myocardial infarction; HF, Heart Failure; CVD, Cardiovascular Disease

Hazard ratios (HRs) and 95% confidence intervals (CIs) were estimated using Cox proportional hazards models. BMI was categorized into tertiles (<25, 25-28.5, ≥28.5 kg/m²). The formal tests for interaction between sLDLR and BMI strata were not statistically significant (all p for interaction >0.05)
